# A microenvironment-adaptive GelMA-ODex@RRHD hydrogel for responsive release of H_2_S in promoted chronic diabetic wound repair

**DOI:** 10.1093/rb/rbae134

**Published:** 2024-11-23

**Authors:** Zhixian Yuan, Wei Zhang, Chang Wang, Chuwei Zhang, Chao Hu, Lu Liu, Lunli Xiang, Shun Yao, Rong Shi, Dejiang Fan, Bibo Ren, Gaoxing Luo, Jun Deng

**Affiliations:** Institute of Burn Research, Southwest Hospital, State Key Lab of Trauma and Chemical Poisoning, Army Medical University (Third Military Medical University), Chongqing 400038, China; Institute of Burn Research, Southwest Hospital, State Key Lab of Trauma and Chemical Poisoning, Army Medical University (Third Military Medical University), Chongqing 400038, China; Institute of Burn Research, Southwest Hospital, State Key Lab of Trauma and Chemical Poisoning, Army Medical University (Third Military Medical University), Chongqing 400038, China; Institute of Burn Research, Southwest Hospital, State Key Lab of Trauma and Chemical Poisoning, Army Medical University (Third Military Medical University), Chongqing 400038, China; Department of Burn and Plastic Surgery, Affiliated Hospital of Nantong University, Nantong 226001, China; Institute of Burn Research, Southwest Hospital, State Key Lab of Trauma and Chemical Poisoning, Army Medical University (Third Military Medical University), Chongqing 400038, China; Institute of Burn Research, Southwest Hospital, State Key Lab of Trauma and Chemical Poisoning, Army Medical University (Third Military Medical University), Chongqing 400038, China; Institute of Burn Research, Southwest Hospital, State Key Lab of Trauma and Chemical Poisoning, Army Medical University (Third Military Medical University), Chongqing 400038, China; Institute of Burn Research, Southwest Hospital, State Key Lab of Trauma and Chemical Poisoning, Army Medical University (Third Military Medical University), Chongqing 400038, China; Institute of Burn Research, Southwest Hospital, State Key Lab of Trauma and Chemical Poisoning, Army Medical University (Third Military Medical University), Chongqing 400038, China; Department of Breast Surgery, Gansu Provincial People's Hospital, Lanzhou, Gansu 730030, China; Institute of Burn Research, Southwest Hospital, State Key Lab of Trauma and Chemical Poisoning, Army Medical University (Third Military Medical University), Chongqing 400038, China; Institute of Burn Research, Southwest Hospital, State Key Lab of Trauma and Chemical Poisoning, Army Medical University (Third Military Medical University), Chongqing 400038, China; Institute of Burn Research, Southwest Hospital, State Key Lab of Trauma and Chemical Poisoning, Army Medical University (Third Military Medical University), Chongqing 400038, China; Institute of Burn Research, Southwest Hospital, State Key Lab of Trauma and Chemical Poisoning, Army Medical University (Third Military Medical University), Chongqing 400038, China

**Keywords:** injectable hydrogel, hydrogen sulfide, wound healing, healing quality, anti-inflammation

## Abstract

Chronic diabetic wounds present significant treatment challenges due to their complex microenvironment, often leading to suboptimal healing outcomes. Hydrogen sulfide (H_2_S), a crucial gaseous signaling molecule, has shown great potential in modulating inflammation, oxidative stress and extracellular matrix remodeling, which are essential for effective wound healing. However, conventional H_2_S delivery systems lack the adaptability required to meet the dynamic demands of different healing stages, thereby limiting their therapeutic efficacy. To address this, we developed an injectable, ROS-responsive H_2_S donor system integrated within a gelatin methacryloyl (GelMA) hydrogel matrix, forming a double-network hydrogel (GelMA-ODex@RRHD). The injectability of this hydrogel allows for minimally invasive application, conforming closely to wound contours and ensuring uniform distribution. The incorporation of oxidatively modified dextran derivatives (ODex) not only preserves biocompatibility but also enables the chemical attachment of ROS-responsive H_2_S donors. The GelMA-ODex@RRHD hydrogel releases H_2_S in response to oxidative stress, optimizing the environment for cell growth, modulating macrophage polarization and supporting vascular regeneration. This innovative material effectively suppresses inflammation during the initial phase, promotes tissue regeneration in the proliferative phase and facilitates controlled matrix remodeling in later stages, ultimately enhancing wound closure and functional recovery. The H_2_S released by GelMA-ODex@RRHD not only expedited the process of wound healing but also improved the biomechanical characteristics of newborn skin in diabetic mice, particularly in terms of stiffness and elasticity. This enhancement resulted in the skin quality being more similar to normal skin during the wound healing process. By aligning therapeutic delivery with the natural healing process, this approach offers a promising pathway toward more effective and personalized treatments for chronic diabetic wounds.

## Graphical abstract

**Figure:**
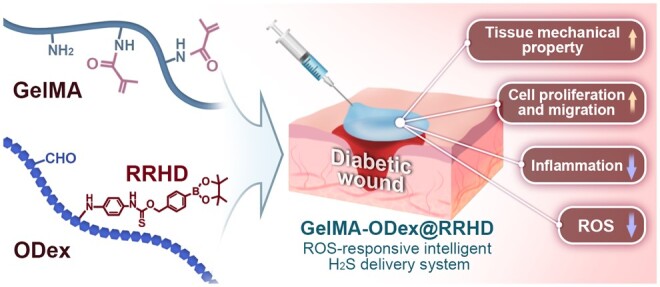

## Introduction

Chronic wounds, a prevalent condition with significant morbidity, impact over 1% of the population, posing a substantial challenge to healthcare systems [1, 2]. Among these, diabetic wounds are particularly concerning, given their high morbidity, mortality and recurrence rates, making them the leading cause of non-traumatic limb amputations globally [3]. Despite advancements in treatments such as surgery [4], negative pressure wound therapy [5], hyperbaric oxygen therapy [6], shock wave therapy [7] and moist wound dressings [8, 9], current interventions are often limited by their focus on specific stages of wound healing [10–12], leading to suboptimal outcomes. The wound microenvironment is inherently complex and dynamic [13], necessitating a full-period controlled treatment approach that offers multi-phase effectiveness and precise regulation.

Although there are many gaseous bioactive molecules, such as NO used in wound treatment with effects on anti-inflammation and promotion of angiogenesis [14, 15]. However, in recent years, it has been found that hydrogen sulfide, as a gaseous signaling molecule, not only has the same effects as other gas signaling molecules but also being able to directly regulate the activity of Matrix metalloproteinases (MMPs), helping to balance the matrix degradation and reconstruction in the process of tissue repair. And compared with NO, H_2_S has lower toxicity [16–18]. Given these properties, the application of H_2_S throughout the entire healing process of chronic diabetic wounds holds significant promise for biomedical innovation. Notably, the biological effects of H_2_S depend on its concentration level. Specifically, lower concentrations of H_2_S under physiological conditions exhibit positive cytoprotective effects. However, once the biosynthetic pathway of H_2_S is blocked or it is applied in excess, the original H_2_S balance in the body is altered, resulting in the reversal of the original stimulatory effect to an inhibitory effect, which has a different impact on physiological homeostasis [19–21]. Studies have shown that appropriate levels of hydrogen sulfide can induce macrophage polarization toward the M2 phenotype and promote the proliferation migration and angiogenesis of human umbilical vein endothelial cells (HUVECs), etc., but the cytotoxicity increased significantly when hydrogen sulfide was treated at a concentration of 2000 μM [22–25]. Several smart H_2_S delivery systems have been developed using biocompatible polymers [26, 27], designed for extended-release and responsiveness to stimuli such as pH [28], light [29] and thiols [30], thereby enhancing the therapeutic efficacy of H_2_S in tissue repair. However, these systems often require stringent release conditions, lacking the flexibility for adaptive, on-demand delivery, which is essential in the fluctuating wound environment [31]. Additionally, the inability of H_2_S to regenerate raises concerns about dose decay or inactivation during the prolonged healing process of diabetic wounds [20]. The demand for H_2_S fluctuates significantly across different healing stages; it is higher during the inflammatory phase due to increased cellular oxidation and consumption, and lower during the proliferative and remodeling phases [18]. Considering the pivotal role of reactive oxygen species (ROS) in chronic wound pathology and the therapeutic importance of H_2_S [32, 33], there is an urgent need to develop a delivery system that can respond to ROS levels, ensuring precise control of H_2_S release to match the specific demands of the repair process. Such a system would not only mitigate the direct damage caused by excessive ROS but also optimize the wound microenvironment, facilitating a smooth transition from the inflammatory phase to the proliferative and remodeling phases.

**Figure 1.**
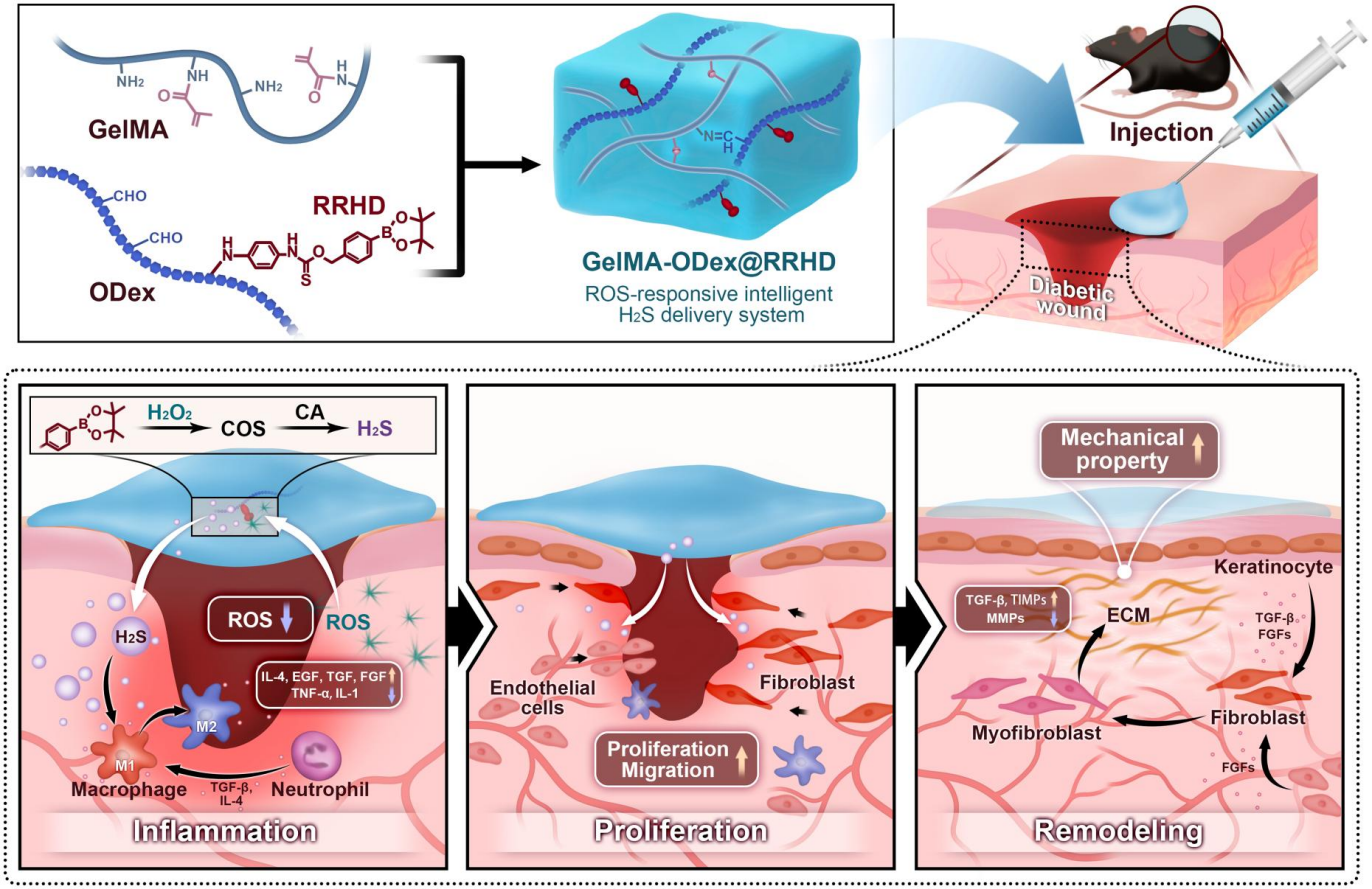
Schematic illustration of the preparation of injectable GelMA-ODex@RRHD hydrogel and its application in repairing full-thickness skin defects in mice.

An innovative therapeutic strategy is proposed through the design of a ROS-responsive intelligent H_2_S donor system aimed at enhancing wound repair as shown in Figure 1. By introducing the oxidatively modified dextran derivative ODex within a GelMA hydrogel matrix, a double-network hydrogel system was formed. The integration of ODex preserved biocompatibility while offering additional pathways for chemical modification, enabling the attachment of ROS-responsive hydrogen sulfide donors to the hydrogel network. As the GelMA concentration increased and ODex was incorporated, the hydrogel structure became denser, with pore sizes optimized for cell growth, all while maintaining sensitivity to fluctuations in ROS levels within the wound microenvironment. Exposure to ROS triggers the hydrogel to release carbonyl sulfide, which is rapidly converted to H_2_S by the ubiquitous enzyme carbonic anhydrase [34], thereby facilitating targeted therapeutic effects at various stages of wound healing. During the initial phase of wound healing, GelMA-ODex@RRHD establishes a continuous H_2_S delivery environment, releasing significant amounts of H_2_S in response to elevated ROS levels. This process effectively curbs the activation of inflammatory pathways, thereby effectively reducing both inflammation and oxidative stress. In the subsequent proliferative phase, H_2_S modulates macrophage polarization from the M1 to the M2 phenotype [35], fostering a pro-healing immune environment. This modulation potentially enhances cell proliferation and migration by releasing adequate levels of H_2_S [26], which also synergistically supports vascular regeneration [36]. As wound recovery progresses to the critical tissue reconstruction and functional recovery phase, the concentration of ROS decreases, and the hydrogel releases lower amounts of H_2_S. This controlled release reduces MMP activity [37], aids in the regulation of extracellular matrix (ECM) remodeling [38] and promotes the maturation of granulation tissue and collagen deposition [39], ultimately leading to stable wound closure and functional recovery. In conclusion, the GelMA-ODex@RRHD material effectively achieves the precise, controlled release of H_2_S, tailored to the varying microenvironments of diabetic wounds during different healing stages, utilizing a ROS-triggered mechanism.

## Materials and methods

### Preparation and characterization of GelMA-ODex@RRHD

#### Synthesis of RRHD

The synthesis steps of ROS-responsive H_2_S donor (RRHD) are shown in Supplementary Figure Sl. In an ice bath, thiophosgene (0.16 ml, 2.04 mmol) was dissolved in 10 ml of anhydrous 1,4-dioxane and added to a solution of phenylboronic acid pinacol ester (234 mg, 1.0 mmol) in 10 ml of 1,4-dioxane for 24 h. After evaporating the solvent, the product was dissolved in 10 ml of dichloromethane. At 0°C, the above solution was added to a solution of p-phenylenediamine (108 mg, 1.0 mmol) in 15 ml of dichloromethane. The mixture was stirred at 0°C for 30 min. The reaction was quenched by adding saturated brine. The mixture was extracted with dichloromethane (3 × 50 ml), and the combined organic layers concentrated under reduced pressure to obtain the crude product. The final product was purified by silica gel column chromatography.^1^H NMR (600 MHz, DMSO-d_6_) *δ* 7.63 (d, *J* = 7.7 Hz, 2H), 7.32 (d, *J* = 7.6 Hz, 2H), 6.35 (s, 4H), 5.24 (t, *J* = 5.7 Hz, 1H), 4.52 (d, *J* = 5.5 Hz, 2H), 1.33–1.22 (m, 12H).

#### Synthesis of GelMA and ODex

In this study, we employed methods based on previously reported work [40, 41], with modifications to optimize the synthesis process of GelMA and ODex and enhance the material's performance for specific applications. Initially, 2.5 g of gelatin was solubilized in a distilled solution at 60°C. Then, 4 ml of MA was incrementally introduced to the reaction, allowing it to react for an additional hour at 50°C with strong agitation. The resultant blend was then dialyzed against distilled water for 45 h at 40°C using a dialysis membrane with a 3500 Da cutoff. Subsequently, the mentioned reaction was lyophilized for future applications. To prepare ODex, firstly, 3.0 g of dextran was mixed in 100 ml of distilled water, and stirred until dissolved. Concurrently, 3.5 g of sodium periodate was put into 25 ml of distilled water under light-protected environment. Upon complete dissolution, the two solutions were combined and agitated in the dark for 3.5 h. To neutralize any remaining sodium periodate, 3 ml of ethylene glycol was gradually added, and the mixture was stirred for an additional hour. The ODex solution was then dialyzed against distilled water for 48 h using a 3500 Da molecular weight cutoff dialysis membrane. The treated ODex solution was then subjected to freezing at −20°C, followed by freeze-drying to yield the ODex. The prepared GelMa and ODex were characterized using Fourier-transform infrared spectroscopy (FTIR).

#### Conjugation of hydrogen sulfide donor molecules to ODex

First, amount of ODex was dissolved in water at 5% (w/v), while RRHD was dissolved in anhydrous DMSO to make a 25 mg/ml solution. Subsequently, the RRHD solution was added dropwise to the ODex solution at room temperature, adjusting the molar ratio of aldehyde groups in ODex to amine groups in RRHD to 1:3, and stirred continuously for 24 h under light-protected conditions. After the reaction, the mixture was transferred to dialyze against water for 48 h. The solution was then collected and lyophilized to get the product.

#### Preparation of GelMA-ODex@RRHD hydrogels

To prepare the hydrogels, GelMA and ODex-RRHD were each dissolved in PBS, and a 0.1% (w/v) concentration of the photoinitiator LAP was added. The solutions were then lighted with 365 nm source to induce gelation. For the gelatin methacryloyl-oxidized dextran@ROS-responsive H_2_S donor (GelMA-ODex@RRHD) hydrogels, several compositions with varying mass fractions of GelMA and ODex@RRHD were synthesized, resulting in formulations named G2OD8, G1OD8 and G0.5OD8. The gelation time of each hydrogel was determined using the bottle inversion technique, where gelation was considered complete when the mixture remained stationary in an upturned centrifuge tube. GelMA-ODex hydrogels were prepared using the same method as described above and were included as an additional group for the biological experiments.

#### The oxidation level of ODex

The degree of oxidation (DO) for ODex is calculated using the following formula [41]: DO = ((*V*_1_ − *V*_0_) * *M* * *M*_W_)/*W* * 100%, where *V*_0_ represents the volume of sodium hydroxide (NaOH) used in the blank experiment, and *V*_1_ is the volume of NaOH consumed in the actual analysis. In this equation, *M* denotes the molarity of the NaOH solution, *M*_W_ is the molecular weight of the glucosyl moiety in the dextran backbone, and *W* is the mass of ODex in grams. The results reported are the mean values derived from analyses of five separate sample batches.

#### Degradation of hydrogels

The *in vitro* degradation characteristics of the G2OD8, G1OD8 and G0.5OD8 were evaluated through the following procedure: initially, in a PBS solution (pH = 7.4) with or without H_2_O_2_ (200 μM) at 37°C [42], the freeze-dried samples were weighed (*W*_0_) and then submerged. At designated intervals, the samples were removed, rinsed multiple times with water, freeze-dried again and their residual weight (*W_t_*) was recorded. The remaining weight percentage (*R*) of the hydrogel was calculated using the equation: *R* = (*W_t_*/*W*_0_) × 100%.

#### Swelling rate test

To test the swelling properties of three types of hydrogels, G2OD8, G1OD8 and G0.5OD8, we immersed samples of each type of hydrogel into phosphate buffer solution (PBS) with the pH set at 7.4. After a preset period, excess water adhering to the hydrogel surface was rapidly absorbed using filter paper, followed by weight measurement of the samples in the wet state (labeled *M_t_*). The swelling rate (*W_t_*) of the sample was calculated according to the following equation: *W_t_* = (*M_t_* − *M*_0_)/*M*_0_ × 100%.

*M*_0_ is the initial dry weight of the hydrogel sample before swelling and *M_t_* is the wet weight after swelling. The swelling test was performed three times for each sample.

#### Time-dependent H_2_S release from hydrogels

As per previously reported work [43], to profile H_2_S release, a NaHS stock solution was first prepared by dissolving 10.0 mg of NaHS in 20.0 ml of deionized water. This solution was then serially diluted with water to concentrations ranging from 5 to 200 μM. For calibrating the H_2_S release profile using the methylene blue assay, 100 μl of each NaHS solution was mixed with 100 μl of a 30 mM FeCl_3_ solution (in 1.2 M HCl) and 100 μl of N, N-dimethyl-p-phenylenediamine. The production of H_2_S from the GelMA-ODex@RRHD hydrogel was initiated by adding 100 μl of the donor's concentrated solution (15 mM, dissolved in DMSO) to a 10 ml PBS solution (pH 7.4, 50 mM) containing 100, 200, 500 μM H_2_O_2_ and 25 μg/ml CA; 200 μM H_2_O_2_ and 10, 25 and 50 μg/ml CA. Samples of 1.0 ml were taken from the reaction mixture at regular intervals and placed into UV–vis spectrometry cuvettes pre-filled with an MB reagent mix, comprising 100 μl zinc acetate (1% w/v), 200 μl N, N-dimethyl-1,4-phenylenediamine sulfate (20 mM in 7.2 M HCl) and 200 μl ferric chloride (30 mM in 1.2 M HCl). The MB reaction was allowed to proceed for 15 min before measuring the absorbance of the solution at 670 nm using a Thermo Evolution 300 UV–Vis spectrophotometer. The concentration of H_2_S in each sample was determined by comparing the absorbance readings to a standard curve generated from known concentrations of NaHS. The results are expressed as mean values ± standard deviation (SD) for a sample size of *n* = 3.

### Cell culture

All cultures were maintained in a standard incubator at 37°C with a humidified 5% CO_2_ atmosphere. To replicate the hyperglycemic conditions characteristic of patients with diabetes, the glucose concentration in all media was adjusted to 25 mM for *in vitro* experiments.

### Therapeutic effects of GelMA-ODex@RRHD *in vitro*

#### Cell compatibility

Calcein-AM/PI were used to evaluate and images were captured using a CLSM; Olympus Corporation, Tokyo 163-0914, Japan.

#### CCK-8 proliferation assay

Cells were seeded at a density of 5 × 10^4^ cells per well in 24-well plates, and cell proliferation was quantified using the CCK-8 cell viability assay, with results normalized to the control group.

#### Cell migration

In 6-well transwell plates, cells were seeded in the lower chamber at a density of 3 × 10^6^ cells per well. Following incubation at 37°C, the cells reached 80–90% confluency. A sterile 1 ml pipette tip was employed to create a vertical scratch on the 6-well transwell plates. Fresh serum-free DMEM or 1640 medium was then added. The scratch area was monitored and photographed using an AX10 inverted fluorescence microscope.

#### Tube formation assay

Matrigel matrix (BD, USA) was thawed at 4°C for 2 h, diluted 1:1 with serum-free DMEM medium and added to pre-cooled 12-well transwell plates (lower chamber) at 400 μl per well. After incubation at 37°C for 1 h, the matrix was allowed to solidify. HUVECs were seeded, following 6 h of incubation at 37°C, images were observed and documented using a fluorescence inverted microscope.

#### Cell imaging of H_2_O_2_-induced H_2_S release from GelMA-ODex@RRHD

RAW 264.7 cells were seeded at 5 × 10^4^ cells per well in a 24-well transwell plate. After 8 h, the cells were treated with DMEM medium containing WSP-1 (50 μM) without FBS for 30 min. Extracellular WSP-1 was removed by washing with PBS, followed by incubation with various concentrations of H_2_O_2_ (0, 25, 50, 100 and 200 μM) in FBS-free DMEM medium and the upper chambers containing pre-prepared GelMA-ODex@RRHD for 30 min. Fluorescence imaging was performed using confocal laser scanning microscopy (CLSM) (Olympus, Japan).

#### The long-term release experiments of H_2_S

Cells were seeded at 5 × 10^4^ cells per well in a 24-well transwell plate incubated with media containing 25 mM glucose. After 8 h, free NaHS (200 μM), GelMA-ODex and GelMA-ODex@RRHD were added to continue the incubation for 72 h. WSP-1 probe was used to detect cellular hydrogen sulfide.

#### Cellular ROS scavenging experiment

Cells were incubated with a DCFH-DA fluorescent probe in the dark at room temperature for 60 min. After washing three times with serum-free medium to remove any unloaded DCFH-DA probe, imaging was conducted using a confocal laser scanning microscope (FV1000, Olympus, Japan).

#### Anti-inflammatory capacity

RAW 264.7 cells were seeded in 24-well plates (5 × 10^4^ cells per well) and incubated for 24 h in an incubator. Then, the media containing H_2_O_2_ (200 μM) were added, and incubated for 24 h. The medium was removed and washed with PBS, RAW 264.7 cells were fixed with 4% paraformaldehyde and immunofluorescence staining was performed to evaluate CD206, iNOS, Arg-1 and CD86.

### Therapeutic effects of GelMA-ODex@RRHD *in vivo*

#### Biocompatibility

The hydrogel was injected into subcutaneous tissue of mice, after 15 days, blood samples were collected for complete blood count and serum biochemistry tests. In addition, mice were euthanized and their hearts, livers, spleens, lungs and kidneys were collected to perform hematoxylin and eosin (H&E) staining.

#### Diabetic mouse model

The animal experiments were carried out according to the Laboratory Animal Administration Rules of China and approved by the Third Military Medical University's Ethics Committee (AMUWEC202117052). C57BL/6 mice were fasted for 12 h with access to water, weighed and then injected intraperitoneally with STZ (100 mg/kg) for 3 consecutive days. One week later, blood glucose levels were measured in tail vein blood using a glucose meter, with mice exhibiting blood glucose concentrations exceeding 16.7 mmol/l classified as having Type I diabetes. A full-thickness skin wound was created using a 6 mm punch. A silicone ring with an inner diameter of 6 mm and an exterior diameter of 10 mm was positioned around the wound as a reference, and the wound was photographed. Immediately post-surgery, the mice were allocated into four groups: the control group (100 μl PBS), GelMA-ODex (100 μl) treated, 100 μl (200 μM) NaHS-treated and GelMA-ODex@RRHD (100 μl) treated. A transparent film dressing (Tegaderm film, USA) was applied to the wound to secure the hydrogel and limit wound contraction due to skin contraction. Dressings were replaced every 3 days. Wound images were captured on days 0, 3, 6, 9 and 12 post-surgery.

#### Histology and immunofluorescence analyses

At various time points post-surgery, mice were euthanized *via* cervical dislocation, and wound tissues were harvested to make paraffin sections, 5 μm thick. H&E staining was performed on sections to evaluate new granulation tissue thickness on day 6, while a Masson's trichrome staining kit (Solarbio, China) was used to assess new collagen tissue formation on day 15. Macrophage polarization in the wound tissue was analysed by immunofluorescence staining for CD206, CD86 and iNOS on day 3. Vascularization and proliferation at the wound site were examined through immunofluorescence staining for CD31 and PCNA on day 6.

#### Measurement of ROS levels in vivo

Fresh frozen sections were washed three times with PBS, stained with dihydroethidium (DHE) at 37°C for 30 min, and sealed with Antifade Mountant containing DAPI.

#### Micromechanical measurements

For micromechanical measurements, full-thickness skin tissue was excised and sectioned into fresh frozen slices from the epidermis to the dermis, each 30 µm thick. These slices were placed on an atomic force microscope (AFM), where the MLCT-C probe was used to measure the mechanical properties of dermis-only slices in a PBS environment.

#### RNA-sequencing and bioinformatics analysis

Wound tissues were harvested 6 days post-treatment and cryopreserved in liquid nitrogen. Total RNA was extracted from the wound tissues using the TaKaRa MiniBEST Universal RNA Extraction Kit (TaKaRa, Japan). Only high-quality RNA samples were selected for library construction. These libraries were pooled and sequenced on the Illumina HiSeq X10 platform (Illumina, USA). Raw data were filtered using fastp software, and clean data were quality-controlled with FastQC software. Upon satisfactory quality control, further analysis was conducted. Differential expression analysis was performed using DESeq2 software, with thresholds set at |fold change| ≥ 2 and *P* values ≤0.05. KEGG pathway enrichment analyses were then carried out based on DEGs.

#### Measurement of inflammatory factor levels

Tissues from mouse wounds were gathered and the levels of IL-1β, IL-4 and TNF-α were measured using enzyme-linked immunosorbent assay (ELISA) following the instructions provided by the manufacturer.

#### Western blotting

Wound tissue was gathered and 200 μl of RIPA lysate was introduced for every 20 mg of tissue. The lysed samples underwent centrifugation at 16 000 *g* for 15 min to collect the supernatant, which included all the proteins. This supernatant was then used for immunoblotting procedures.

### Statistical analysis

Statistical analyses were conducted using GraphPad Software 10.1. Non-normally distributed variables were analysed by Kruskal–Wallis test. Analysis for normally distributed variables was performed using one-way, two-way analysis (ANOVA) or *t*-tests. All data are expressed as means ± SD (∗*P* < 0.05, ∗∗*P* < 0.01, ∗∗∗*P* < 0.001, ∗∗∗∗*P* < 0.0001).

The details regarding reagents used are provided in the Supplementary Material.

## Results and discussions

### Synthesis and characterization of GelMA-ODex@RRHD

**Figure 2.**
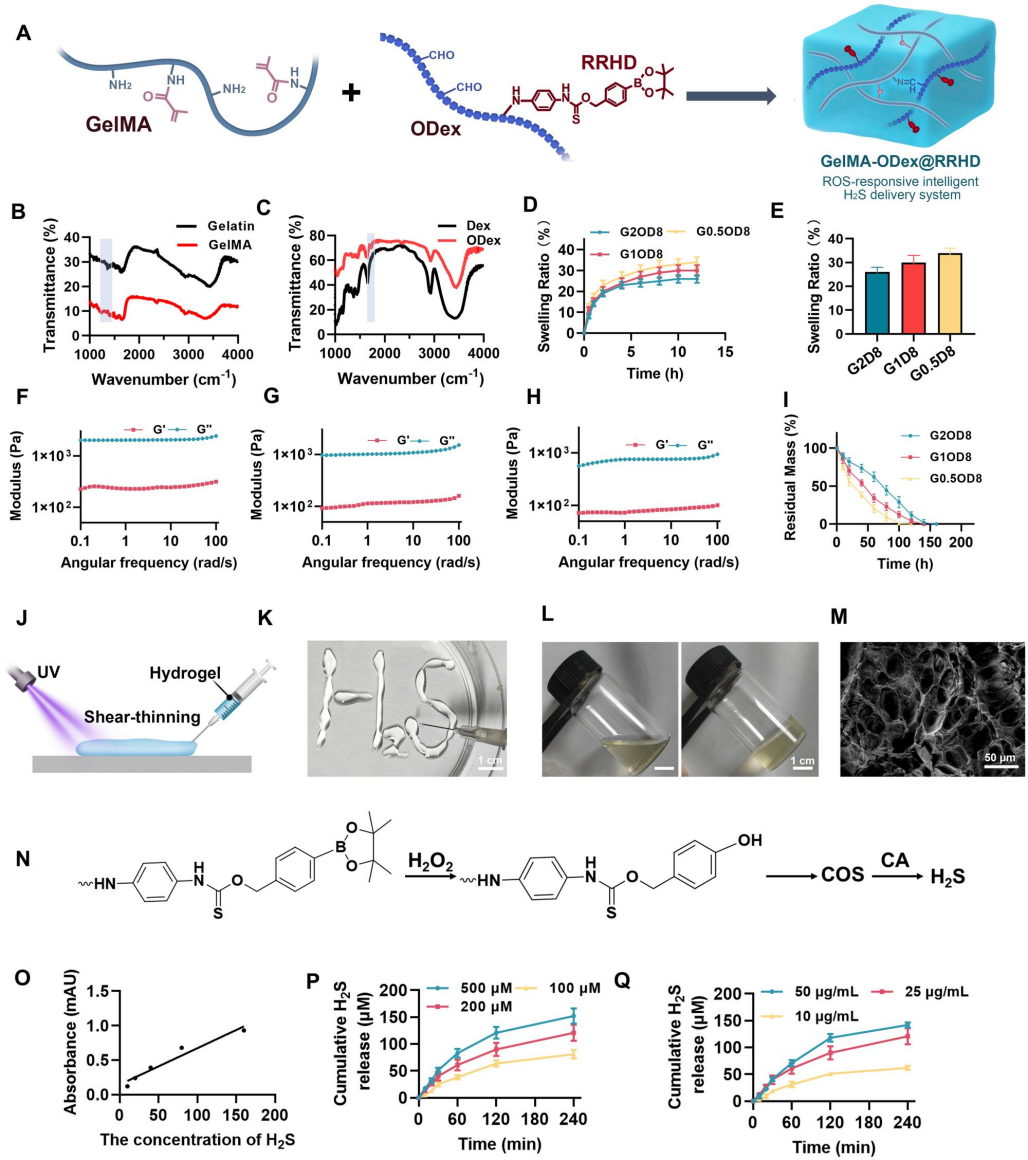
Characterization of GelMA-ODex@RRHD. (**A**) Schematic illustration of the preparation of GelMA-ODex@RRHD hydrogel materials. (**B**) Fourier-transform infrared (FTIR) spectra of gelatin and GelMA. (**C**) FTIR spectra of dextran (DEX) and ODex. (**D**) Swelling data of three different ratios of GelMA and ODex hydrogels (G2OD8, G1OD8, G0.5OD8). (**E**) Statistical analysis of the swelling data shown in Figure **D** at 12h, comparing the swelling behavior of the three hydrogels. The storage modulus (*G*′) and loss modulus (*G*″) of G2OD8 (**F**), G1OD8 (**G**) and G0.5OD8 (**H**) hydrogel as a function of angular frequency. (**I**) Degradation profile of the G2OD8, G1OD8, G0.5OD8 with H_2_O_2_ (*n* = 5). (**J**) Illustration of the injectability of the hydrogel. (**K**) Pattern of H_2_S before light exposure, demonstrating the injectability of the hydrogel. (**L**) Changes in the gel before and after light exposure, with the left image showing the gel before light exposure and the right image showing the gel after solidification under light. (**M**) Scanning electron microscopy (SEM) image of GelMA-ODex@RRHD hydrogel. (**N**) Mechanism diagram of ROS-responsive H_2_S generation, illustrating the chemical reactions that release H_2_S in the presence of H_2_O_2_. (**O**) Standard curve of H_2_S release, showing the absorbance of different H_2_S concentrations. (**P**) H_2_S release profiles at different H_2_O_2_ concentrations (25 μg/ml CA, *n* = 5). (**Q**) H_2_S release profiles at different CA concentrations (200 μM H_2_O_2_, *n* = 5).

The successful synthesis of the RRHD monomer was confirmed by high-resolution mass spectrometry (HRMS) and ^1^H NMR. The spectrum showed a prominent peak at *m*/*z* 407.1589 (Supplementary Figure S2), which corresponds to the [M + Na] ^+^ adduct, where M represents the RRHD monomer. Additionally, the ^1^H NMR spectrum showed the expected chemical shifts, further verifying the structure of the RRHD monomer (Supplementary Figure S3). Following synthesis, the RRHD monomer was conjugated with ODex *via* its reactive amino groups. Subsequently, the remaining aldehyde groups in ODex reacted with GelMA, followed by photoinitiated crosslinking to form the final hydrogel (Figure 2A). GelMA is highly regarded in tissue engineering, functioning as versatile biomaterials with applications ranging from tissue adhesives to scaffolds for tissue regeneration [44]. The FTIR analysis of the synthesized GelMA revealed a characteristic absorption peak around 1020 cm^−1^, which is associated with the –CH_2_ group’s torsional vibration (Figure 2B). However, the inherent mechanical and adhesive limitations of GelMA hydrogels have restricted their broader application. To address this issue, our research introduced a second interpenetrating polymer network into the GelMA hydrogel formulation, enhancing its performance. The FTIR spectra were utilized to examine the chemical structure of ODex, identifying a stretching vibration peak of the C=O group of saturated aldehydes near 1690 cm^−1^ (Figure 2C). This peak confirms the successful oxidation of dextran's hydroxyl groups to aldehydes *via* sodium periodate. The oxidation degree of ODex was determined to be 97.72 ± 0.48% in our study. The introduction of aldehyde functional groups allowed ODex to maintain dextran’s biocompatibility while providing additional avenues for chemical modification. This ODex-modified adhesive has been applied in wound repair. Besides, our findings indicate that GelMA content and ODex incorporation influence the hydrogel's swelling behavior (Figure 2D). The highest swelling rate observed was in G0.5OD8 (34.01 ± 2.89%), while G2OD8 displayed the lowest (26.19 ± 0.59%) at 12 h (Figure 2E). However, there is no obvious significant difference in the swelling rate among these three groups. Nonetheless, a lower swelling rate is more conducive to wound healing [45]. Additionally, the rheological properties of the hydrogels were tested (Figure 2F–H). The loss modulus (*G*″) of the hydrogels was consistently lower than the storage modulus (*G*′), which remained stable over time, suggesting that the hydrogels achieved full crosslinking. This enhancement in modulus is attributed to the higher GelMA concentration and the addition of ODex. Furthermore, as strain increased, the storage modulus (*G*′) of all three scaffolds exhibited a decreasing trend, which is advantageous for filling irregular wounds. Our study also investigated the degradation behavior of three different hydrogel formulations: G2OD8, G1OD8 and G0.5OD8. These hydrogels differ primarily in their crosslinking density, which significantly influences their degradation rates. The G2OD8 hydrogel exhibited the slowest degradation rate, requiring nearly 200 h without H_2_O_2_ (Supplementary Figure S4). Besides, the G2OD8 hydrogel also exhibited the slowest degradation rate under H_2_O_2_, requiring nearly 160 h for complete degradation (Figure 2I). This degradation rate is advantageous for the stable and sustained release of hydrogen sulfide from the hydrogel promoting better healing outcomes. Based on the above findings, G2OD8 was selected as the final formulation for GelMA-ODex@RRHD due to its superior performance. Besides, Figure 2J illustrates the state of the injectable hydrogel before and after photo-crosslinking. In Figure 2K, the hydrogel is successfully extruded into the letters “H_2_S” using a syringe, demonstrating its excellent injectability and fluidity. The figure contrasts the hydrogel's condition before and after light exposure: the left image shows the hydrogel in its liquid state prior to exposure, while the right image depicts its transformation into a solid gel following exposure to 365 nm light. This rapid solidification into a stable structure underscores the hydrogel's capability for quick gelation upon light exposure, which is advantageous for clinical applications, enabling the hydrogel to provide immediate protection and support once injected into the wound site. After incorporating ODex into the GelMA hydrogel matrix, a subtle yellow tint was observed, likely resulting from the Schiff base reaction (Figure 2L). Scanning electron microscopy (SEM) analysis revealed the microstructure of these hydrogels, with pore diameters ranging from 30 to 70 μm, which are conducive to cell adhesion and growth (Figure 2M). This porous structure not only promotes cell proliferation but also facilitates nutrient exchange, supporting cellular activities during the wound repair process.

### The H_2_S release profile of GelMA-ODex@RRHD

The performance and regulatory mechanisms of the H_2_S release system are further explored. The H_2_S responsive release mechanism, depicted in Figure 2N, begins with the oxidation of phenylboronic acid pinacol ester by H_2_O_2_, converting it into phenolic hydroxyl groups. A subsequent 1,6-elimination reaction produces 4-fluoroaniline, 4-hydroxybenzyl alcohol and carbonyl sulfide (COS), which is then hydrolyzed by CA to generate H_2_S. The hydrogen sulfide standard curve (Figure 2O) demonstrates a strong linear relationship, providing a reliable quantitative basis for further experiments. The study of H_2_S release kinetics revealed significant influences of both H_2_O_2_ concentration and carbonic anhydrase (CA) concentration on the release process. Under a fixed CA concentration (25 μg/ml), H_2_S release significantly increased with higher H_2_O_2_ concentrations, with the 500 μM H_2_O_2_ group achieving a cumulative release of nearly 150 μM within 4 h, compared to less than 100 μM in the 100 μM H_2_O_2_ group (Figure 2P). Similarly, at a fixed H_2_O_2_ concentration (200 μM), higher CA concentrations enhanced both the rate and cumulative amount of H_2_S release, with the 50 μg/ml CA group releasing close to 140 μM H_2_S in 4 h, while the 10 μg/ml CA group only about 50 μM H_2_S (Figure 2Q). Higher concentrations of both H_2_O_2_ and CA resulted in increased hydrogen sulfide production. Additionally, introducing dynamic non-covalent bonds within the material enhances its ability to adapt to changes in the skin microenvironment. These bonds, such as hydrogen bonds and ionic interactions, allow the material to respond flexibly under varying oxidative stress conditions. When the wound environment exhibits higher levels of ROS, these dynamic bonds facilitate structural adjustments in the material, optimizing the release of H_2_S. Similar strategies have already proven effective in other gel materials [44–46]. These interactions help achieve more sensitive H_2_S release, aligning with the varying needs at different stages of the wound healing process. Besides, by precisely modulating the concentrations of H_2_O_2_ and CA, it is possible to achieve controlled H_2_S release kinetics, thereby developing intelligent materials tailored to the specific needs of different stages in wound repair.

Excellent biocompatibility is a crucial prerequisite for hydrogel applications. The results of cell live/dead staining showed that there was no significant change in the cell death rate after the application of GelMA-ODex@RRHD from the control group (*P* > 0.05) (Supplementary Figure S5A). The histopathological and blood biochemical indicators of the major organs in the mice treated with GelMA-ODex@RRHD did not show any abnormalities (*P* > 0.05) (Supplementary Figure S5B and C). Relevant literature shows that H_2_O_2_ is the main component of ROS in wounds [47], and wound fluid contains micromolar concentrations of steady-state hydrogen peroxide [48]. In a mouse model of total excisional wounds, the level of hydrogen peroxide in wounds peaked at approximately 200 μM during the inflammatory phase (day 2) and declined to 150 μM on day 5 [42]. Therefore, we utilized 200 μM H_2_O_2_ in response to different volumes of GelMA-ODex@RRHD [42]. We evaluated the changes in cell viability under co-culture with cells in different volumes of GelMA-ODex@RRHD hydrogel with the help of CCK-8 kits. The experimental results showed that GelMA-ODex@RRHD had no significant cytotoxicity, and cell viability was gradually enhanced with increasing hydrogel dose in the range of 0–50 μl hydrogel volume showing that GelMA-ODex@RRHD also had a role in promoting cell proliferation. We hypothesized this might be because the amount of hydrogen sulfide initially released from the low-dose GelMA-ODex@RRHD hydrogel was not sufficient to cope with the demands of the high oxidative stress environment, and thus an increase in the administered dose was required. Further observations revealed that cell viability was maximized when the hydrogel volume reached approximately 50 μl (Supplementary Figure S6A). To further investigate whether elevated ROS levels would lead to excessive H_2_S production and cause cytotoxicity, we also investigated the changes in cell viability under 500 μM H_2_O_2_ conditions and obtained similar results (Supplementary Figure S6B). Through the above experiments, we ensured the safety and effectiveness of the hydrogel in the application.

### *In vitro* release of H_2_S

**Figure 3.**
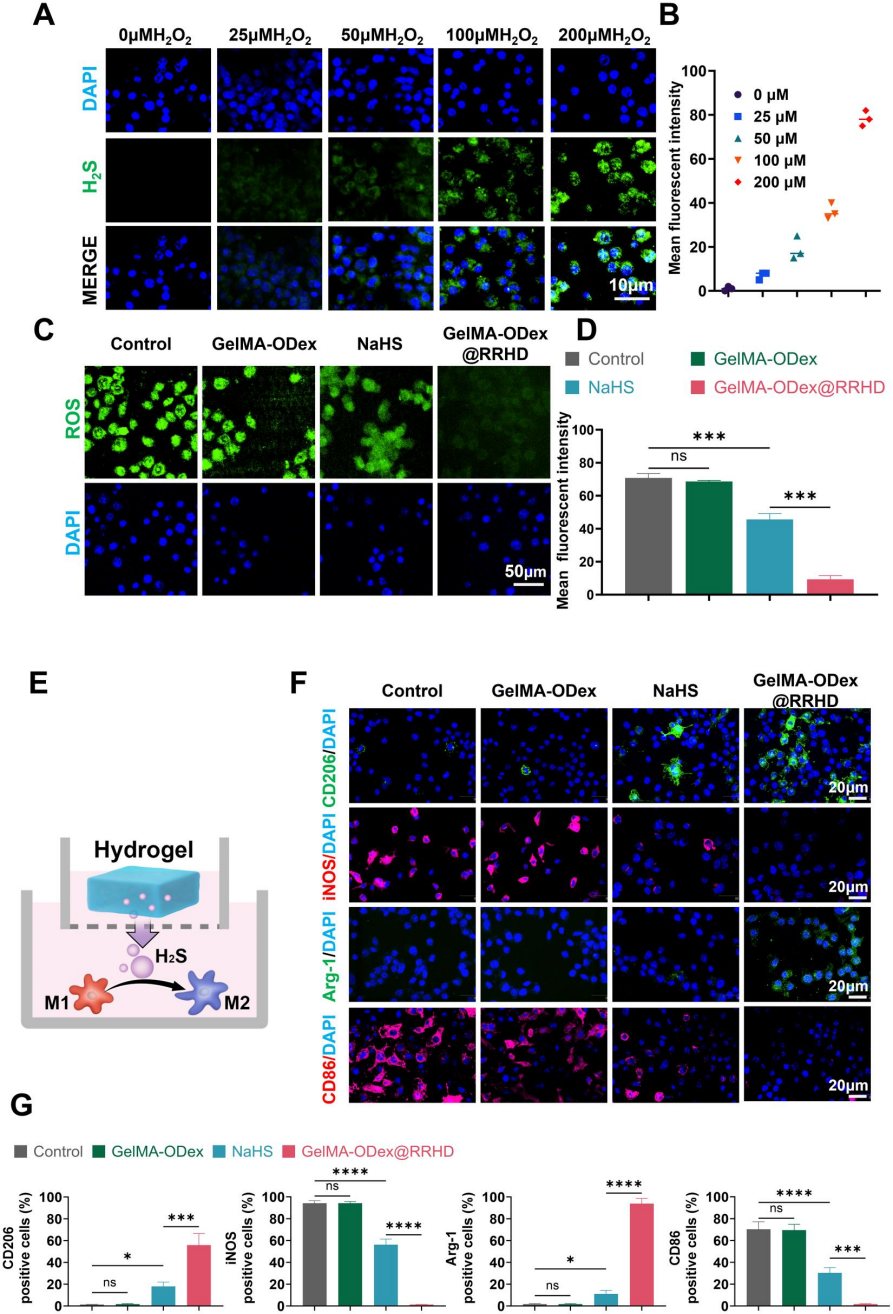
GelMA-ODex@RRHD altered M1 macrophages switching to M2 phenotype *in vitro*. (**A**, **B**) Representative images and quantitative analysis of the release of H_2_S in response to H_2_O_2_ in RAW264.7 stained by WSP-1 probe. (**C**, **D**) Representative images and quantitative analysis of ROS scavenging ability in RAW264.7 stained by DCFH-DA probe. (**E**) Schematic representation of macrophage polarization by GelMA-ODex@RRHD. (**F**) Representative images of CD206, iNOS, Arg-1 and CD86 immunofluorescence staining of RAW 264.7. (**G**) Statistical data of the percentage of CD206+, iNOS+, Arg-1+ and CD86+ macrophages. (*n* = 3; mean ± SD; ns, not statistically significant; *P < 0.05, **P < 0.01, ***P < 0.001, ****P < 0.0001).

WSP-1 was utilized to monitor H_2_S accumulation in RAW264.7 cells (Figure 3A). The absence of fluorescence in the control group indicated that GelMA-ODex@RRHD did not release H_2_S under normal cellular conditions. However, the addition of H_2_O_2_ resulted in detectable fluorescence, suggesting that ROS can activate GelMA-ODex@RRHD to release H_2_S within the cellular environment. Moreover, the fluorescence intensity increased in a dose-dependent manner with rising H_2_O_2_ levels, indicating that higher ROS concentrations can induce greater H_2_S release (Figure 3B).

### Anti-inflammatory effects *in vitro*

To assess whether GelMA-ODex@RRHD has superior anti-inflammatory effects, we simulated mouse wound H_2_O_2_ levels with H_2_O_2_ (200 μM). H_2_O_2_ induced polarization of M0 phenotypes to M1 phenotypes. The expression of CD86 (M1 marker), iNOS (M1 marker), Arg-1 (M2 marker) and CD206 (M2 marker) was identified by immunofluorescence staining to confirm the polarization status under different conditions. Confocal laser scanning microscopy revealed that, compared to the control group, the NaHS group showed a significant increase in CD206+ (*P* < 0.05) and Arg-1+ (*P* < 0.05) macrophages, along with a reduction in CD86+ (*P* < 0.0001) and iNOS+ (*P* < 0.0001) macrophages, indicating that H_2_S release may have enhanced M2 activation. Furthermore, compared to the NaHS group, GelMA-ODex@RRHD-treated macrophages exhibited significantly higher expression of CD206 (*P* < 0.001) and Arg-1 (*P* < 0.0001), along with lower expression of CD86 (*P* < 0.001) and iNOS (*P* < 0.0001), further validating that GelMA-ODex@RRHD continuously promotes the conversion of M1 macrophages to M2 macrophages through sustained H_2_S release in response to ROS (Figure 3E–G).

Diabetic wounds are characterized by elevated levels of ROS and persistent oxidative stress [49]. After 12 h of treatment, the intracellular ROS fluorescence intensity in the GelMA-ODex@RRHD group was significantly lower than both the control and GelMA-ODex groups. A similar effect was observed with 200 μM NaHS treatment; however, the fluorescence intensity in the GelMA-ODex@RRHD group was even lower than that in the NaHS-treated group (*P* < 0.0001) (Figure 3C and D).

### GelMA-ODex@RRHD stimulates cell proliferation, migration and angiogenesis *in vitro*

**Figure 4.**
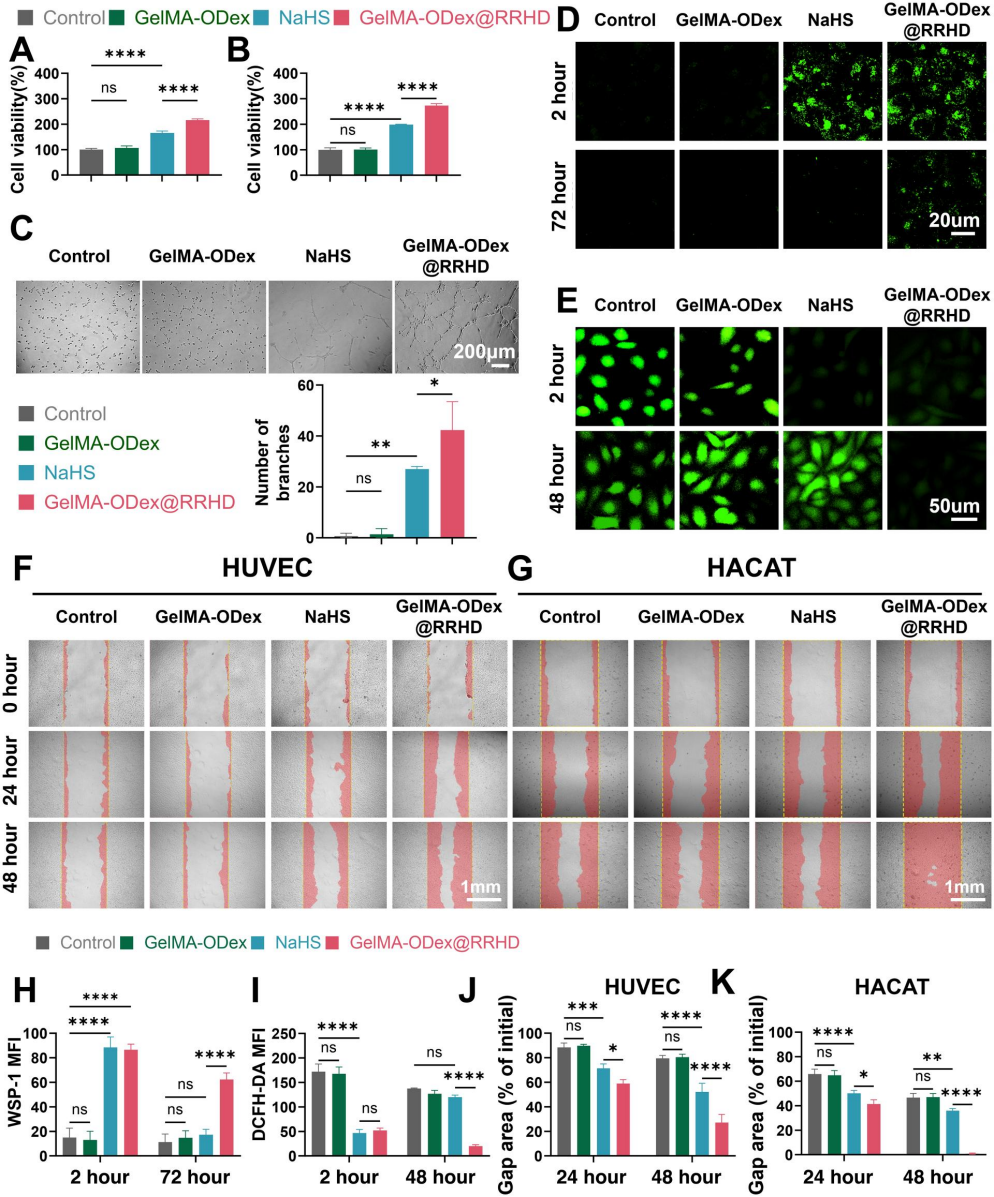
Effects of GelMA-ODex@RRHD on HUVECs and HACATs proliferation and migration. (**A**, **B**) Effect on the proliferation of HUVECs and HACATs cultured for 2 days. (**C**) Representative images and quantitative analysis of tube formation assay in HUVECs. (**D**, **H**) Representative images and quantitative analysis of H_2_S detection in HUVECs. (**E**, **I**) ROS scavenging ability in HUVECs. (**F**, **J**) Images and quantification of the migration of HUVECs. (**G**, **K**) Images and quantification of the migration of HACATs. (*n* = 3; mean ± SD; ns, not statistically significant; *P < 0.05, **P < 0.01, ***P < 0.001, ****P < 0.0001).

First, we determined the optimal H_2_S concentration for cell treatment using a CCK-8 kit. The experimental data showed that NaHS treatment significantly promoted the growth of cell numbers, especially at NaHS concentrations of about 200 μM, where cell viability peaked and then slightly decreased but maintained at a level higher than that of the control group (Supplementary Figure S7). Therefore, we chose 200 μM NaHS to treat these cells. Based on these findings, the effect of GelMA-ODex@RRHD on HUVECs and HaCaTs was further investigated. The Cell Counting Kit-8 (CCK-8) assay revealed that within 48 h, the viability of HUVECs and HaCaT cells was significantly enhanced within 48 h following treatment with NaHS and GelMA-ODex@RRHD. Cells exposed to the sustained-release H_2_S from GelMA-ODex@RRHD showed significantly higher viability than those exposed to the rapidly released H_2_S from NaHS (*P* < 0.0001) (Figure 4A and B). The effect of GelMA-ODex@RRHD on cell migration was assessed using scratch assays (Figure 4F and G). Compared to the control and GelMA-ODex@RR groups, the addition of GelMA-ODex@RRHD and NaHS promoted the migration of HUVECs and keratinocytes, accelerating the closure of cell-free gaps. Moreover, cells treated with GelMA-ODex@RRHD not only exhibited a significantly faster gap closure rate at 24 h compared to those treated with free NaHS, but this difference became even more pronounced at 48 h (Figure 4J and K). The H_2_S-specific fluorescent probe WSP-1 was used to assess intracellular H_2_S levels. At 2 h, groups treated with GelMA-ODex@RRHD and NaHS exhibited obvious green H_2_S fluorescence. At 72 h, only GelMA-ODex@RRHD maintained evident green H_2_S fluorescence, with other groups showing nearly no fluorescence (Figure 4D and H). In summary, GelMA-ODex@RRHD supports HUVEC and keratinocyte proliferation by continuously releasing H_2_S. Similarly, in a high-glucose environment, the intracellular ROS fluorescence intensity of HUVECs cultured with GelMA-ODex@RRHD and NaHS decreased at 2 h. However, by 48 h, the fluorescence intensity in the NaHS group was not significantly different from that of the control and GelMA-ODex groups (*P* > 0.05) (Figure 4E and I), indicating that NaHS cannot sustain H_2_S release to continuously clear ROS. The angiogenic capacity of HUVECs was evaluated through matrigel tube formation assays, revealing that the number of branches was substantially higher in GelMA-ODex@RRHD-treated HUVECs than in NaHS-treated HUVECs after 6 h (*P* < 0.05) (Figure 4C). These results suggest that the enhanced angiogenesis observed in various *in vitro* assays is a consequence of the sustained H_2_S release by GelMA-ODex@RRHD.

### GelMA-ODex@RRHD promotes diabetic wound healing

**Figure 5.**
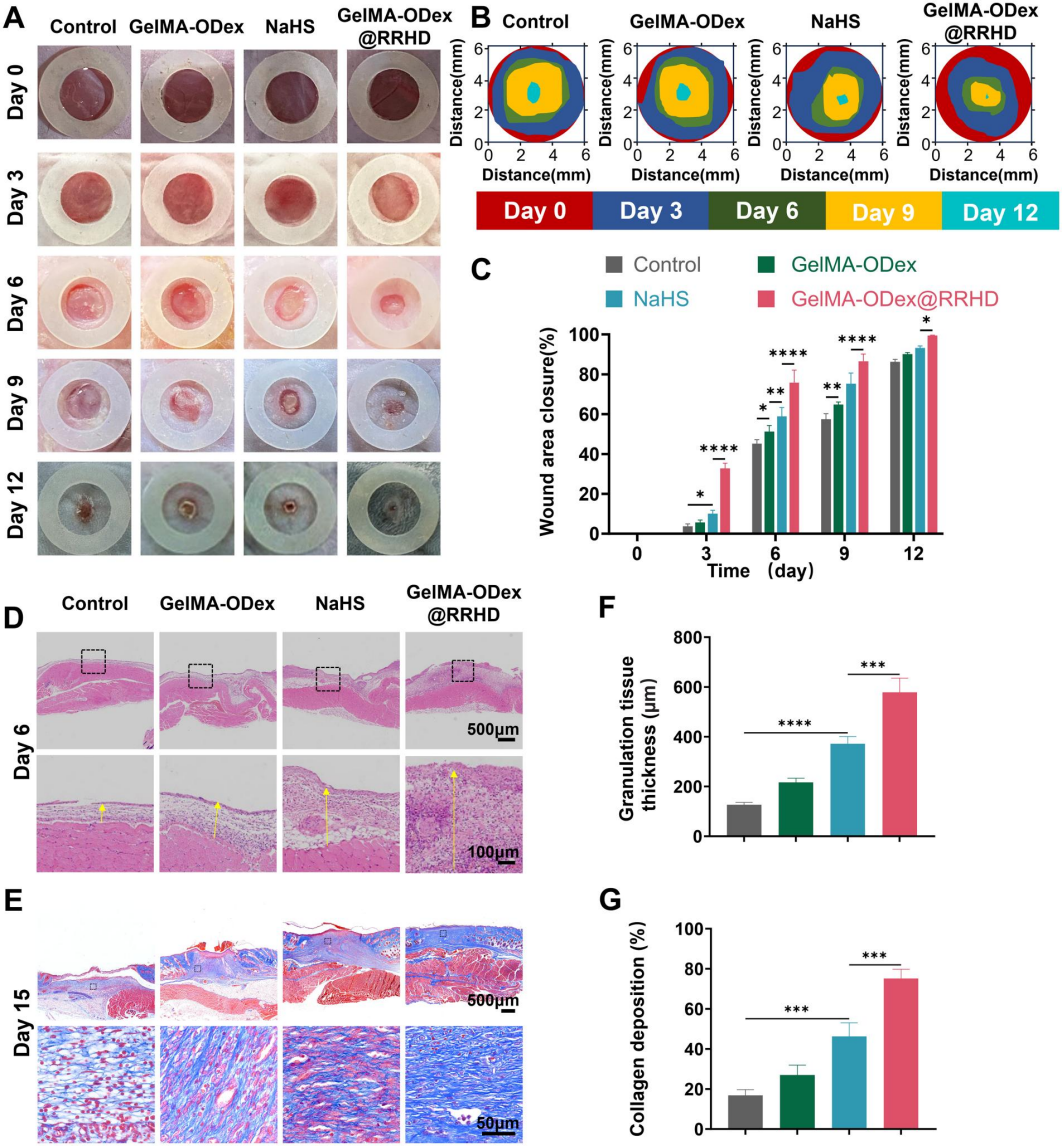
GelMA-ODex@RRHD accelerated diabetic wound repair and tissue regeneration *in vivo*. (**A**) Representative digital images of the wound area on days 0, 3, 6, 9 and 12. (**B**) Fractions of wounds healed by different treatments on days 3, 6, 9 and 12. (**C**) Quantitative analysis of wound area closure for each group. (**D**) H&E staining of the wound area reflected the regenerated skin on day 6. (**E**) Representative images of collagen deposition stained with Masson on day 15. (**F**) Quantitative determination of the thickness of the new granulation tissue on day 6 after surgery. (**G**) The total collagen expression on day 15 was quantified by ImageJ software. (*n* = 3; mean ± SD; ns, not statistically significant; *P < 0.05, **P < 0.01, ***P < 0.001, ****P < 0.0001).

Wound sizes in the NaHS and GelMA-ODex@RRHD groups were notably smaller than those in the control group. Importantly, compared to the rapid-release hydrogen sulfide donor NaHS, wounds treated with GelMA-ODex@RRHD exhibited significantly faster healing on days 3, 6, 9 and 12 (Figure 5A–C). H&E staining on day 6 post-surgery demonstrated that granulation tissue formation was significantly improved in the GelMA-ODex@RRHD group compared to the control group (Figure 5D and F). Masson's trichrome staining on day 15 post-surgery revealed enhanced collagen deposition and composition in the NaHS group compared to the control group (Figure 5E and G).

**Figure 6.**
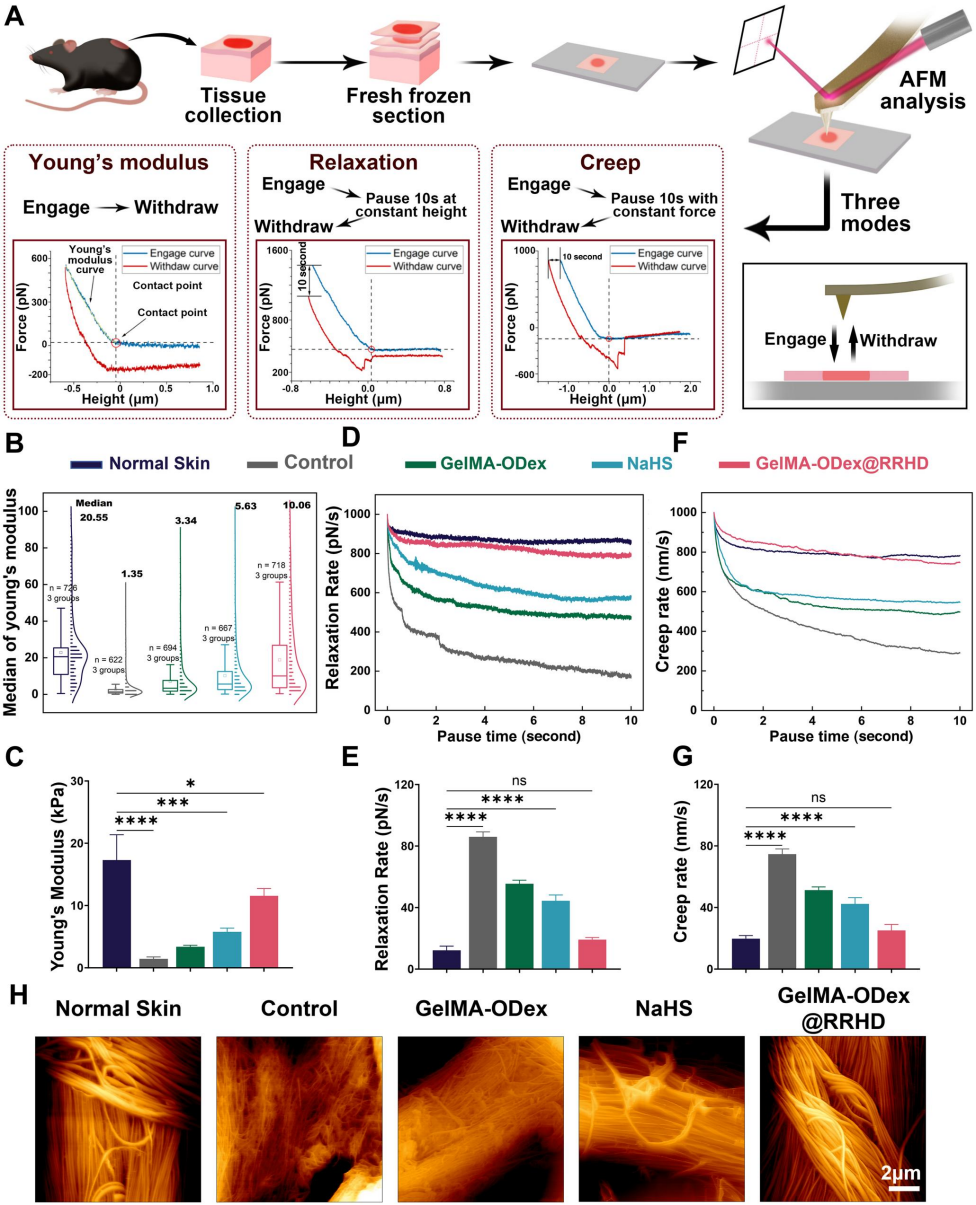
GelMA-ODex@RRHD improved the biomechanical characteristics of newborn skin. (**A**) Atomic force microscope and force curve for different tests. (**B**) Numerical distribution of Young's modulus. (**C**) The statistical analysis of Young's modulus values (*n* = 3/group). (**D**) Time-dependent stress relaxation curves. (**E**) The statistical analysis of relaxation rates (*n* = 3). (**F**) Time-dependent strain creep curves. (**G**) The statistical analysis of creep rates (*n* = 3). (**H**) Representative digital images of the new collagen by AFM. (ns, not statistically significant; *P < 0.05, **P < 0.01, ***P < 0.001, ****P < 0.0001).

Atomic force microscopy (AFM) analysis has emerged as a pivotal tool in elucidating the intricate relationship between material intervention and the enhancement of biomechanical properties in diabetic mouse skin (Figure 6A) [50]. This comprehensive approach to both the speed and quality of healing represents a unique advantage of our material compared to others [51]. Quantitative analysis of Young's modulus demonstrated substantial increases in neo-dermal skin treated with these materials. Remarkably, GelMA-ODex@RRHD treatment exhibited the most significant improvement, with a 10.11 KPa increase in Young's modulus, indicative of a substantial strengthening of neo-dermal skin (Figure 6B and C).

Moreover, relaxation and creep tests, conducted alongside indentation tests, further highlighted the positive impact of these materials on the resilience of neo-dermal tissues. These tests revealed a significant reduction in the relaxation and creep rates of skin treated with GelMA-ODex, NaHS and GelMA-ODex@RRHD. The most substantial reduction in relaxation rate was observed in GelMA-ODex@RRHD-treated skin, with a decline of 66.8 pN/s, signifying a marked improvement in the tissue's ability to withstand deformation and maintain its integrity (Figure 6D and E). The creep rate also showed a significant decrease across all three materials. Specifically, the most pronounced reduction was observed in GelMA-ODex@RRHD-treated skin, with a decrease of 54.8 nm/s, underscoring this material's exceptional ability to confer long-term stability and resilience to neo-dermal tissue (Figure 6F and G). Furthermore, the AFM scans of the injured tissue revealed that the collagen fiber arrangement in the GelMA-ODex@RRHD group closely resembled that of the healthy skin, with a rougher texture and collagen fibers that were more undulating (Figure 6H).

Collectively, these results highlight the potential of H_2_S in enhancing the biomechanical properties of diabetic mouse skin, thus improving the quality of skin post-wound healing. The substantial improvements in both stiffness and resilience, as evidenced by AFM-based indentation, relaxation and creep tests, underscore the promising therapeutic implications of these materials for enhancing wound healing outcomes in patients with diabetes.

**Figure 7.**
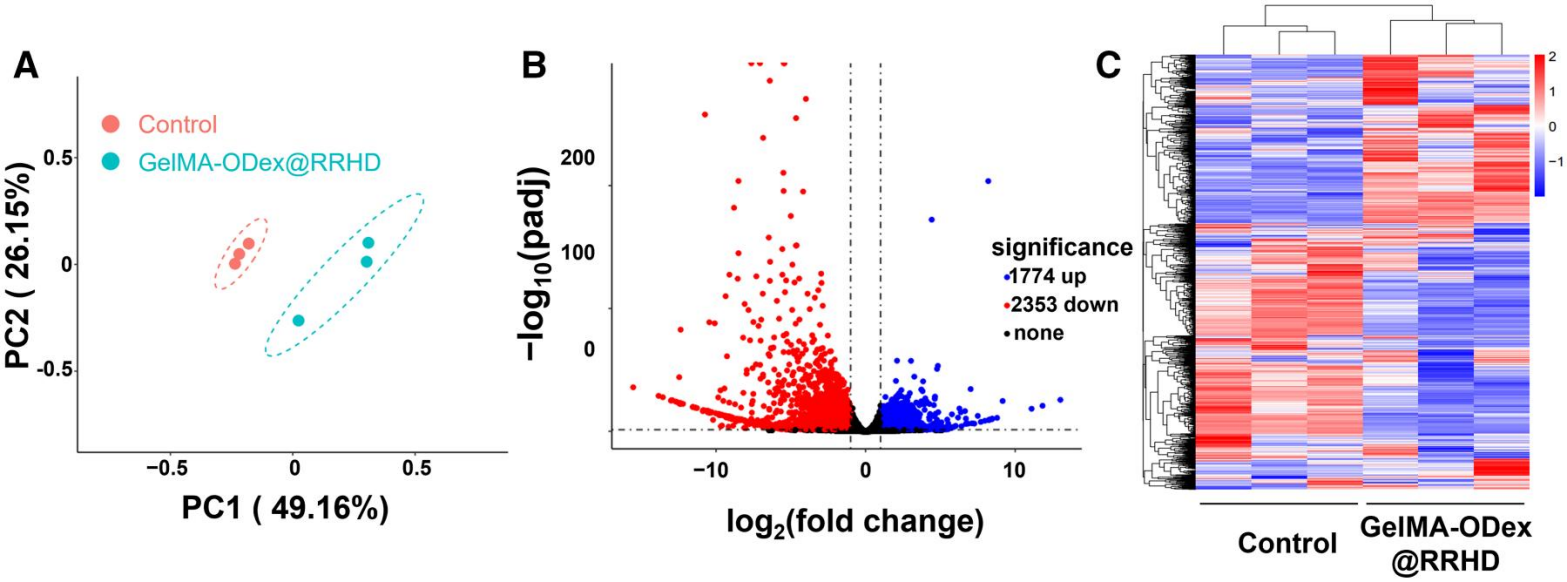
Global assessments of the diabetic wound microenvironment after treatment with GelMA-ODex@RRHD using RNA-seq. (**A**) Principal component analysis (PCA) was performed based on differentially expressed genes (DEGs) in the wound tissue of the two groups. (**B**) Volcano plots showing the upregulated and downregulated genes in response to GelMA-ODex@RRHD treatment. (**C**) Heat map of all genes in the diabetic wound microenvironment after GelMA-ODex@RRHD. (*n* = 3).

Six days post-treatment, the wounds transitioned from the inflammatory phase to the proliferation and remodeling phase. To evaluate the microenvironment of diabetic wounds, wound tissue samples were collected for RNA-sequencing (RNA-seq) analysis. Unguided principal component analysis (PCA) revealed significant disparities in transcriptome profiles between the control group and the GelMA-ODex@RRHD-treated group (Figure 7A). Following GelMA-ODex@RRHD treatment, the empirical Bayes method identified 4127 genes that were significantly differentially expressed between the two groups, with 1774 upregulated and 2353 downregulated, as depicted in the volcano plot (Figure 7B) (fold change ≥2; *P* < 0.05). Hierarchical clustering analysis further illustrated the differential gene expression between the wound tissues of the control and GelMA-ODex@RRHD-treated mice (Figure 7C).

**Figure 8.**
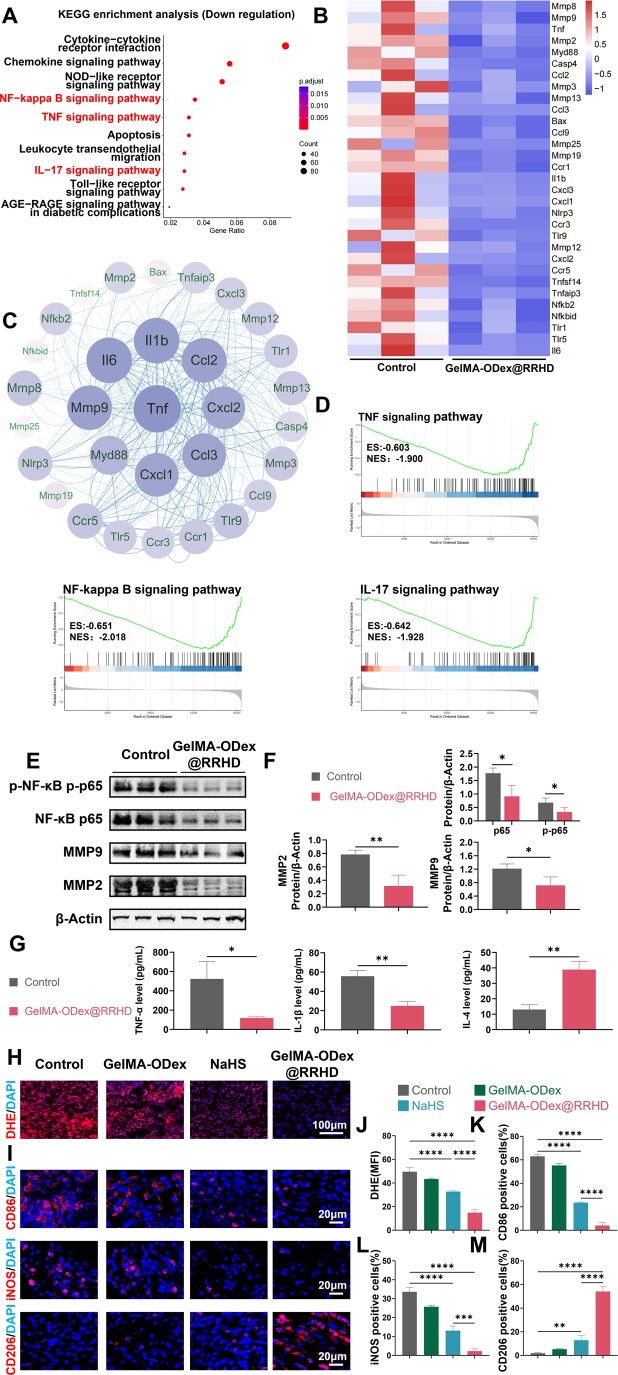
Effects of GelMA-ODex@RRHD on ROS clear and macrophage polarization *in vivo*. (**A**) KEGG pathways enrichment analysis of the downregulated genes. (**B**) Heat map of downregulated genes (fold change ≥ 2 and *P* < 0.05). (**C**) Protein–protein interaction network of downregulated genes. (**D**) Downregulated KEGG pathway GSEA. (**E**) Western blot results of protein expression of NF-κB, p-NF-κB, MMP2 and MMP9 in three individual skin wound tissues 6 days after the operation. (**F**) Quantification of western blot analysis for NF-κB, p-NF-κB, MMP2 and MMP9. (**G**) Expression of TNF-α, IL-1β and IL-4 after 6 days of treatment. (**H**, **J**) Representative confocal images and quantification analysis of the fluorescence assay of ROS in the wound tissues stained by DHE probe on day 3. (**I**) Representative images of CD86, iNOS and CD206 immunofluorescence staining on day 3. (**K**, **L**, **M**) Statistical data of the percentage of CD86+, iNOS+ and CD206+ macrophages. (*n* = 3; mean ± SD; ns, not statistically significant; *P < 0.05, **P < 0.01, ***P < 0.001, ****P < 0.0001).

Kyoto Encyclopedia of Genes and Genomes (KEGG) pathway enrichment analysis of downregulated genes indicated that GelMA-ODex@RRHD treatment effectively inhibited pathways associated with chronic wound inflammation, including IL-17, TNF and NF-κB signaling pathways (Figure 8A). Gene set enrichment analysis (GSEA) of the gene expression data for KEGG enrichment corroborated these findings (Figure 8D).

As shown in Figure 8B, genes related to wound inflammation (TNF, IL-6, CCL3, CCL4 and CXCL1) and proteolysis (MMP8, MMP2 and MMP3) were significantly downregulated. These results suggest that GelMA-ODex@RRHD mitigates inflammatory responses, promotes cell migration and reduces apoptosis by downregulating these cytokines, thereby diminishing the activation of genes involved in inflammatory and necrotic cascade reactions. This, in turn, ameliorates the inflammatory microenvironment of diabetic wounds. Additionally, enzyme-linked immunosorbent assay (ELISA) results indicated that GelMA-ODex@RRHD treatment significantly reduced the expression of proinflammatory factors TNF-α and IL-1β, while increasing the expression of the anti-inflammatory factor IL-10 (Figure 8G).

Concurrently, protein–protein interaction (PPI) network analysis was conducted using the dataset (Figure 8C). Elevated TNF-α levels inhibit critical aspects of wound healing, including cell proliferation, collagen synthesis and angiogenesis, and may also exacerbate inflammatory responses, thus delaying the healing process. In diabetic inflammatory wounds, IL-6 may affect the number, activity and secretion of immune cells, thereby impacting wound repair and healing [52]. MMP2 and MMP9, both members of the MMP family, are gelatinases capable of degrading various ECM components [53]. During wound healing, the ECM not only facilitates the migration and attachment of endothelial cells and the formation of granulation tissue but also influences cell survival. GelMA-ODex@RRHD-treated wounds exhibited reduced expression of MMP2 and MMP9 compared to the control group, indicating that GelMA-ODex@RRHD suppressed excessive ECM degradation, thereby promoting wound healing. Western blot analysis corroborated the results of the transcriptomic analysis (Figure 8E and F).

The inflammatory response in chronic diabetic wounds is driven by ROS [54]. DHE fluorescence staining on day 3 revealed that wounds treated with GelMA-ODex@RRHD exhibited a markedly higher ROS scavenging rate than those treated with NaHS (*P* < 0.0001) (Figure 8H and J). This result aligns with the *in vitro* ROS scavenging experiments (Figure 2D). Immunofluorescence staining demonstrated that CD206 expression was most pronounced in the GelMA-ODex@RRHD group. Simultaneously, compared to the control group, the expression of CD86 and iNOS was significantly downregulated in the GelMA-ODex, NaHS and GelMA-ODex@RRHD groups (Figure 8I, K, L, and M).

**Figure 9.**
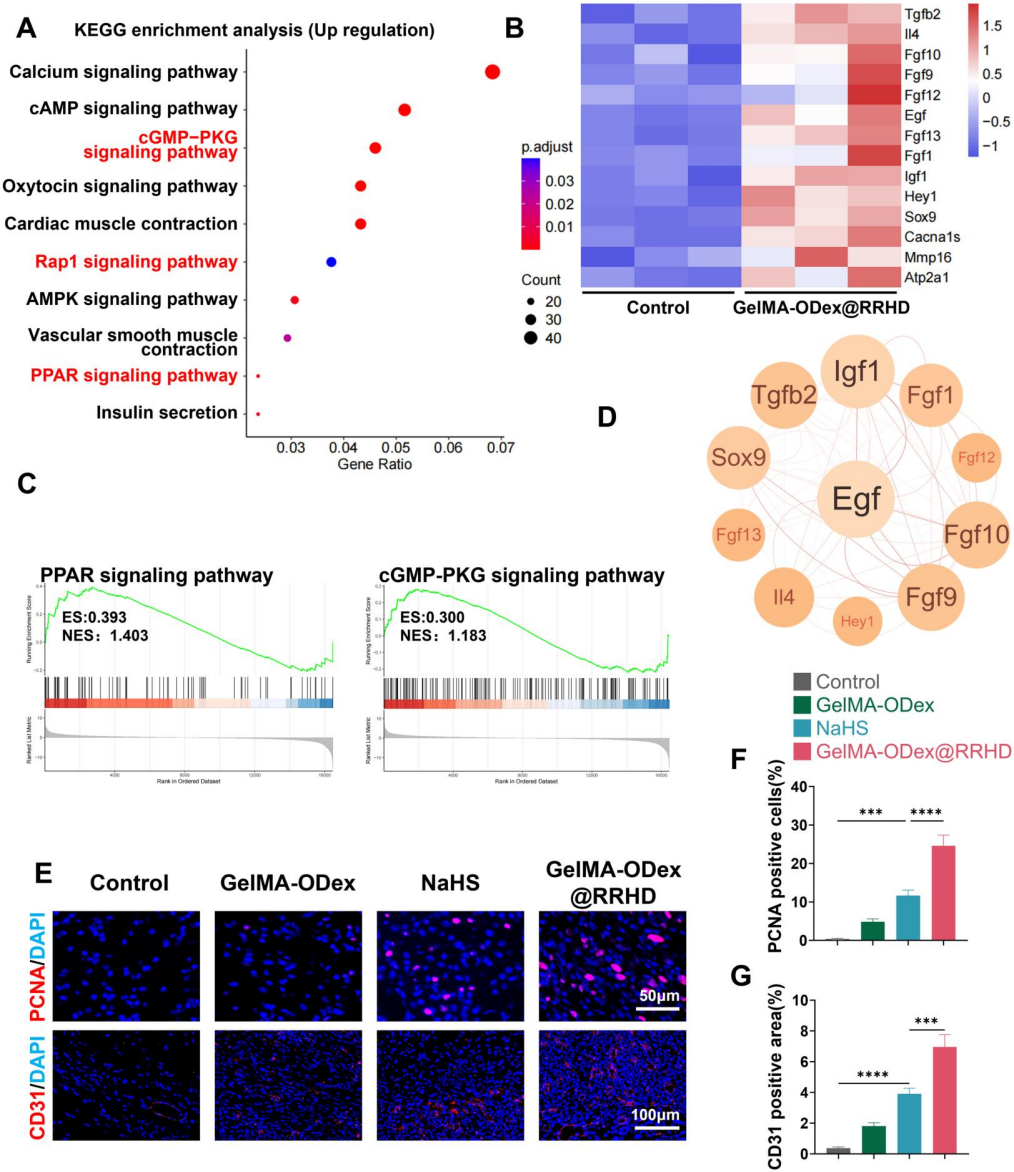
Effects of GelMA-ODex@RRHD on cell proliferation and angiogenesis *in vivo*. (**A**) KEGG pathways enrichment analysis of the upregulated genes. (**B**) Heat map of upregulated genes (fold change ≥ 2 and *P* < 0.05). (**C**) Upregulated KEGG pathway GSEA. (**D**) Protein–protein interaction network of downregulated genes. (**E**) Representative images of PCNA and CD31 immunofluorescence on day 6. (**F**) Statistical data of the percentage of PCNA+ cells. (**G**) Statistical data of CD31 positive area in the wound tissue. (*n* = 3; mean ± SD; ns, not statistically significant; *P < 0.05, **P < 0.01, ***P < 0.001, ****P < 0.0001).

KEGG pathway enrichment analysis of upregulated genes revealed that GelMA-ODex@RRHD treatment effectively activated pathways associated with cell adhesion, migration, proliferation and survival, such as the Rap1, cGMP-PKG and PPAR signaling pathways (Figure 9A). GSEA of the gene expression data corroborated these findings (Figure 9C).

The expression of genes related to wound healing (EGF, TGFβ2) and the anti-inflammatory cytokine IL-4 was significantly upregulated (Figure 9B and D). IL-4 and TGFβ2 are cytokines that promote tissue regeneration, wound healing, nerve fiber regrowth and M2 macrophage polarization [55, 56]. Upregulated FGF1 promotes endothelial cell proliferation and tubular structure formation, directly supporting angiogenesis. Activation of upregulated proteins demonstrated that GelMA-ODex@RRHD significantly promotes cell proliferation and migration, accelerating wound closure, epithelial regeneration and wound tensile strength while increasing ECM deposition and promoting angiogenesis.

Vascularization was assessed on day 6 using immunofluorescence labeling of CD31. A significant increase in CD31-positive areas, indicative of vascular structures, was observed in the GelMA-ODex@RRHD group (*P* < 0.0001) (Figure 9E and F). Proliferating cell nuclear antigen (PCNA), a reliable marker of cell proliferation [57], was also evaluated in wound samples on day 6 to assess overall cellular proliferation. Immunostaining results showed a significant increase in PCNA expression in the GelMA-ODex@RRHD group compared to the other groups (Figure 9E and G).

## Conclusion

This study successfully developed GelMA-ODex@RRHD, an innovative ROS-responsive intelligent H_2_S delivery system for treating diabetic wounds. This double-network hydrogel, integrating GelMA and ODex, exhibits superior performance in controlled H_2_S release, ROS scavenging, anti-inflammatory effects, macrophage polarization, cell proliferation and migration, as well as matrix deposition and tissue remodeling. Notably, our study introduces the novel concept that H_2_S released by GelMA-ODex@RRHD not only accelerates the wound healing process but also enhances the biomechanical properties of diabetic mouse skin, thereby improving skin quality post-healing. These results position GelMA-ODex@RRHD as a promising therapeutic approach for diabetic wound management, offering a dynamic and targeted solution that addresses multiple facets of the wound healing process. By providing sustained and controlled H_2_S release in response to the wound environment, GelMA-ODex@RRHD represents a significant advancement in chronic wound care, paving the way for more effective treatments in this challenging field.

## Supplementary Material

rbae134_Supplementary_Data
