# Supplementary material for: A microenvironment-adaptive GelMA-ODex@RRHD hydrogel for responsive release of H2S in promoted chronic diabetic wound repair
Source: Regen Biomater. 2024 Nov 23;12:rbae134. doi: 10.1093/rb/rbae134 (PMC11703554; doi:10.1093/rb/rbae134)
Supplement: rbae134_Supplementary_Data [file rbae134_supplementary_data.docx]

***Supplementary materials***

**A Microenvironment-Adaptive GelMA-ODex@RRHD Hydrogel for Responsive Release of H2S in Promoted Chronic Diabetic Wound Repair**

Zhixian Yuan^a, 1^, Wei Zhang^a, 1^, Chang Wang^a^, Chuwei Zhang^a, b^, Chao Hu^a^, Lu Liu^a^, Lunli Xiang^a^, Shun Yao^a^, Rong Shi^a, c^, Dejiang Fan^a^, Bibo Ren^a,^ *, Gaoxing Luo^a,^ *, Jun Deng^a,^ *

^a^ Institute of Burn Research, Southwest Hospital, State Key Lab of Trauma and Chemical Poisoning, Army Medical University (Third Military Medical University), Chongqing, 400038, China.

^b^ Department of Burn and Plastic Surgery, Affiliated Hospital of Nantong University, 20 Xisi Road, Nantong 226001, China

^c^ Department of Breast Surgery, Gansu Provincial People's Hospital, Lanzhou, Gansu 730030, China

* Correspondence address. E-mail: [Djun88@Tmmu.edu.cn](mailto:Djun88@Tmmu.edu.cn), [djun.123@163.com](mailto:djun.123@163.com) (J.D.); [logxw@Tmmu.edu.cn](mailto:logxw@Tmmu.edu.cn), [logxw@hotmail.com](mailto:logxw@hotmail.com) (G.L.); [renmoshuang@163.com](mailto:renmoshuang@163.com) (B.R.)

^1^ These authors contributed equally to this work.

**MATERIALS AND REAGENTS**

Thiophosgene (Sigma-Aldrich, USA); phenylboronic acid pinacol ester (Huaxia Chemical, Chengdu, China); anhydrous 1,4-dioxane (Sigma-Aldrich, USA); p-phenylenediamine (Huaxia Chemical, Chengdu, China); anhydrous dichloromethane (Fisher Scientific, USA); gelatin (Huaxia Chemical, Chengdu, China); methacrylic anhydride (Aladdin, Shanghai, China); dextran (Aladdin, Shanghai, China); trifluoroacetic acid (Aladdin, Shanghai, China); lithium phenyl-2,4,6-trimethylbenzoylphosphinate (LAP) (Sigma-Aldrich, USA)Roswell Park Memorial Institute (RPMI) medium 1640, Dulbecco’s modified Eagle’s medium, fetal bovine serum (FBS), penicillin-streptomycin solution, and trypsin were obtained from Gibco (Grand Island, NY, USA). Streptozotocin (STZ) and dihydroethidium (DHE, ≥95%) were acquired from Sigma-Aldrich (St. Louis, MO, USA). Modified Masson's Trichrome Stain Kit (G1346) and the Cell Counting Kit-8 (CA1210) were purchased from Beijing Solarbio. 2,7-dichlorodihydrofluorescein diacetate (DCFH-DA, S0033S), and Calcein/PI Cell Viability/Cytotoxicity Assay Kit (C2015S)were purchased from Beyotime. Washington State Probe-1(WSP-1) was from Shanghai MKbio. CD86 Polyclonal Antibody, Invitrogen (Product # MA1-10299), Dilution 1:1000; CD206 Polyclonal Antibody, Proteintech (Product #18704-1-AP) , Dilution 1:1000; β-actin Recombinant antibody Proteintech (Product # 81115-1-RR) , Dilution 1:10000;Anti-Mannose Receptor antibody, Abcam (Product # ab64693), Dilution 1:1000; Anti-iNOS antibody, Abcam (Product #ab178945), Dilution 1:1000; Anti-Liver Arginase antibody, Abcam ( Product #ab96183), Dilution 1:1000; Mouse momoclonal AntiCD31 antibody, Abcam (Product # ab182981), Dilution 1:1000; [Anti-PCNA antibody, Abcam](https://www.abcam.cn/products/primary-antibodies/pcna-antibody-epr3821-ab92552.html) (Product # ab92552), Dilution 1:1000; Donkey Anti-Rabbit IgG H&L (Alexa Fluor® 647) preadsorbed, , Abcam (Product #ab150063), Dilution 1:2000; Donkey Anti-Rabbit IgG H&L (Alexa Fluor® 594) preadsorbed, Abcam (Product #ab150064), Dilution 1:2000; Anti-MMP2 antibody, Abcam (Product #ab92536) , Dilution 1:1000; Anti-MMP9 antibody, Abcam (Product #ab283575), Dilution 1:1000; [Anti-NF-kB p65 antibody, Abcam (Product #ab32536)](https://www.abcam.cn/products/primary-antibodies/nf-kb-p65-antibody-e379-ab32536.html), Dilution 1:2000; [Anti-NF-kB p65, Abcam](https://www.abcam.cn/products/primary-antibodies/nf-kb-p65-phospho-s536-antibody-ep2294y-bsa-and-azide-free-ab239882.html) [(Product #ab239882)](https://www.abcam.cn/products/primary-antibodies/nf-kb-p65-phospho-s536-antibody-ep2294y-bsa-and-azide-free-ab239882.html), Dilution 1:1000. Mouse TNF-α ELISA Kit（Servicebio, Wuhan, China）, mouse IL-4 ELISA Kit（Servicebio, Wuhan, China）and mouse IL-1β ELISA Kit（Servicebio, Wuhan, China）; Masson’s Trichrome Stain Kit（Solarbio， Beijing， China）， DMEM culture medium(Gibco, American), Transwell culture plate (Corning, USA), Cell culture plate(Corning, USA).

Male C57BL/6 mice (aged 8-10 weeks, 18-20 g, clean grade) and male Sprague–Dawley rats were bought from the Animal Centre of the Army Medical University (Third Military Medical University, TMMU). All animal experiments were conducted following protocols approved by the TMMU Experimental Animal Welfare and Ethics Committee.

Human umbilical vein endothelial cells (HUVECs, cat. no. PCS-100-010) and mouse monocyte macrophage leukemia cells (RAW 264.7, cat. no. TIB-71) were obtained from the American Type Culture Collection (ATCC). Immortalized keratinocytes (HaCaT) were provided by the Chinese Academy of Sciences (Shanghai, China).

**
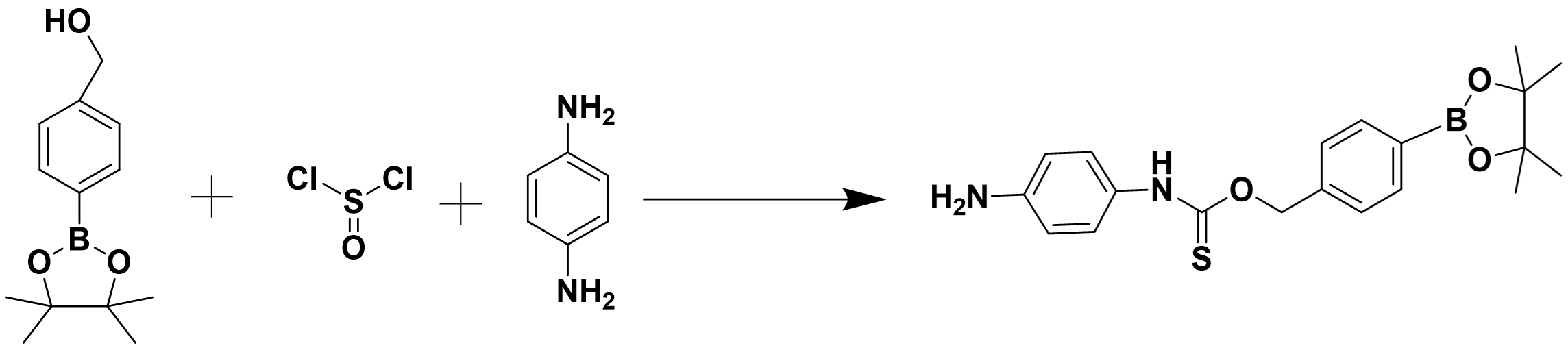
**

Figure S1. Synthesis Scheme of ROS-Responsive H₂S Donor (RRHD)

**
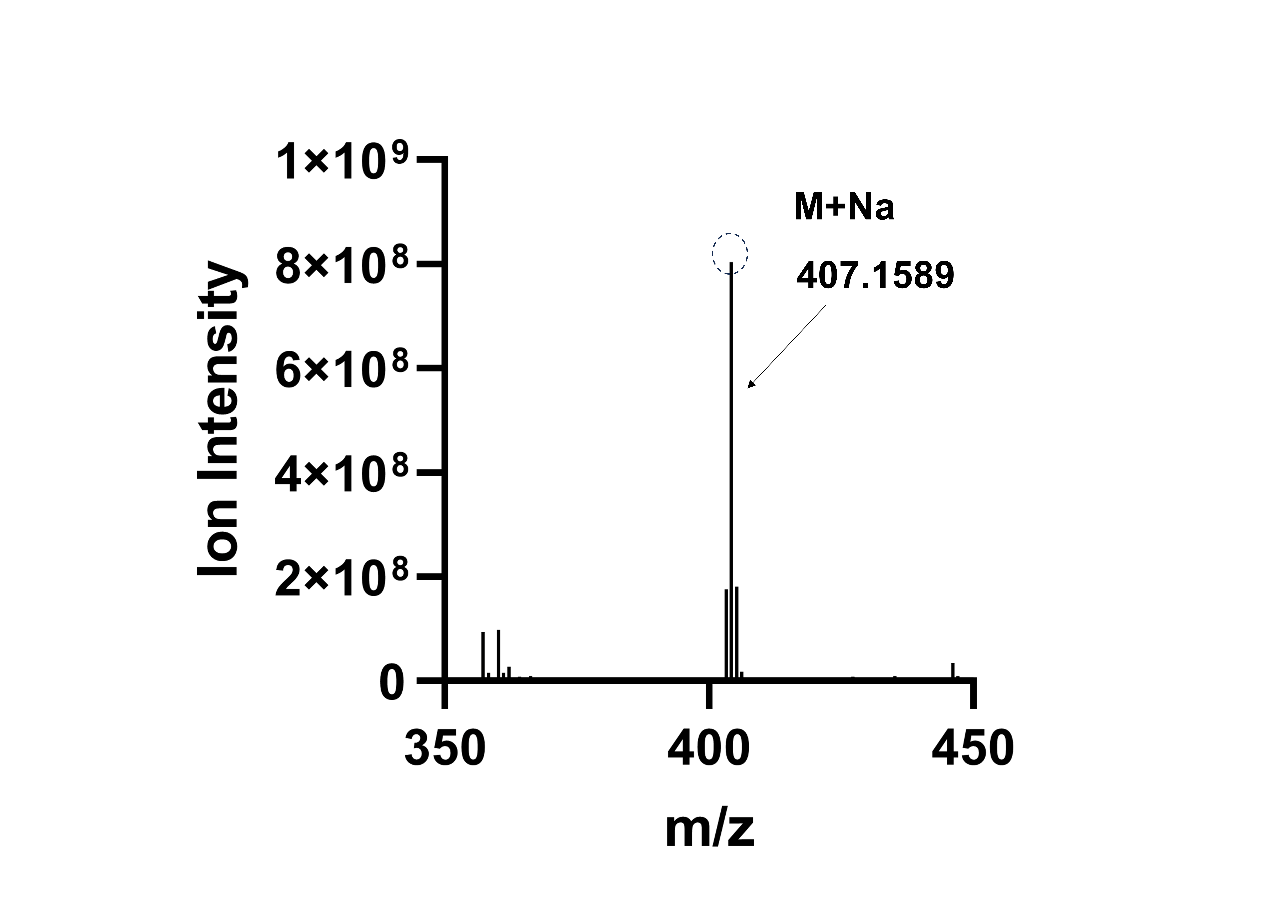
**

Figure S2. HRMS of the RRHD

**
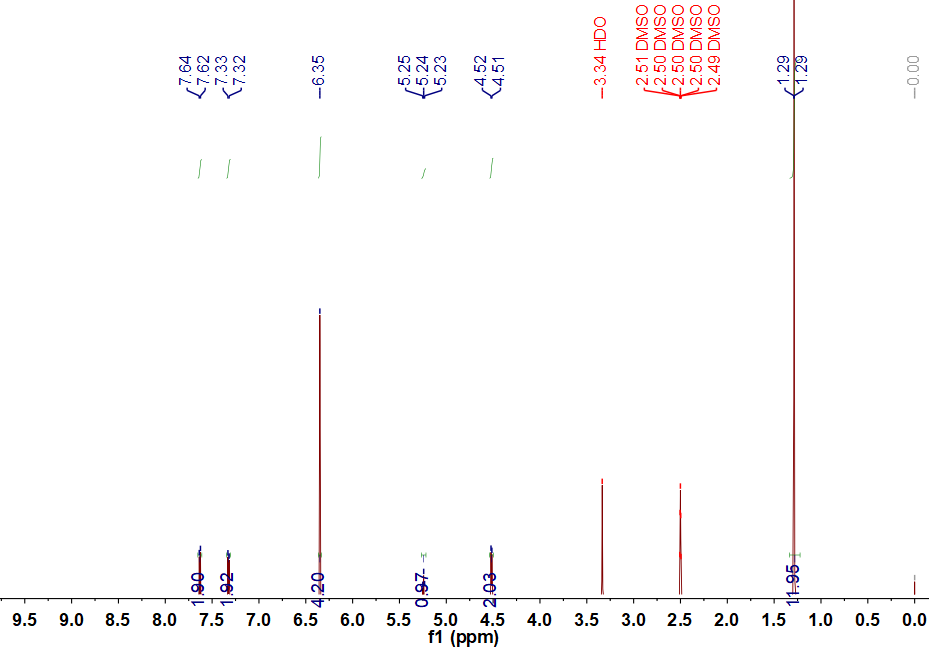
**

Figure S3. ^1^H NMR of the RRHD

**
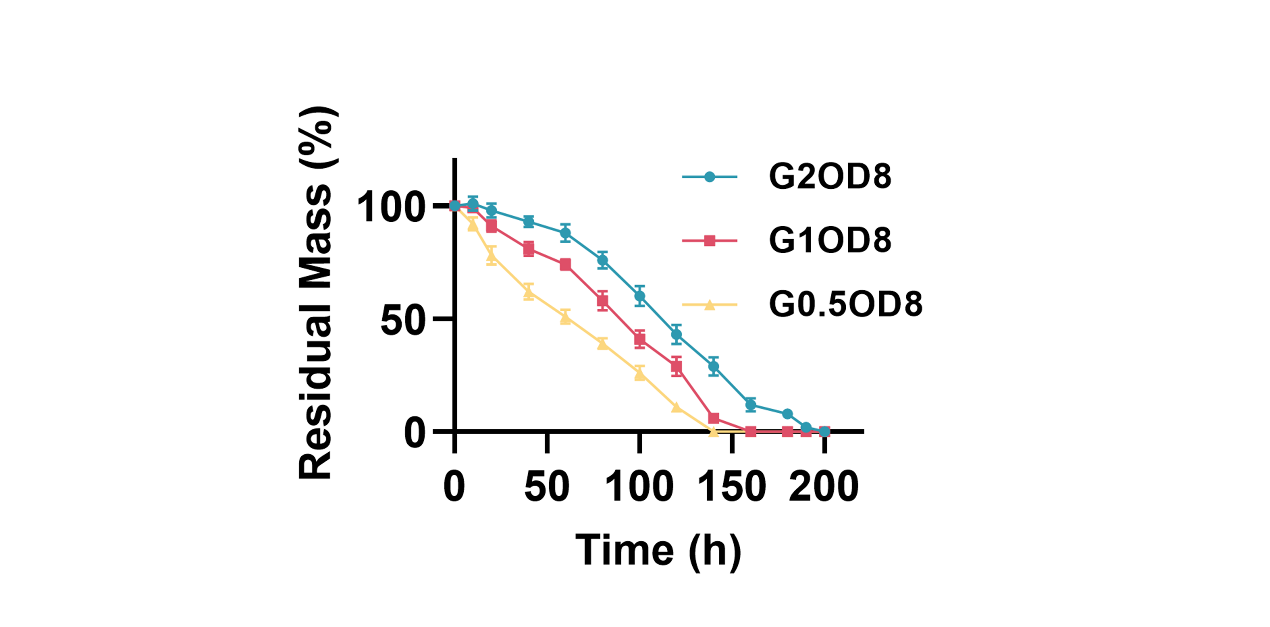
**

Figure S4. Degradation profile of the G2OD8, G1OD8, G0.5OD8 without H_2_O_2_ (n=5).

**
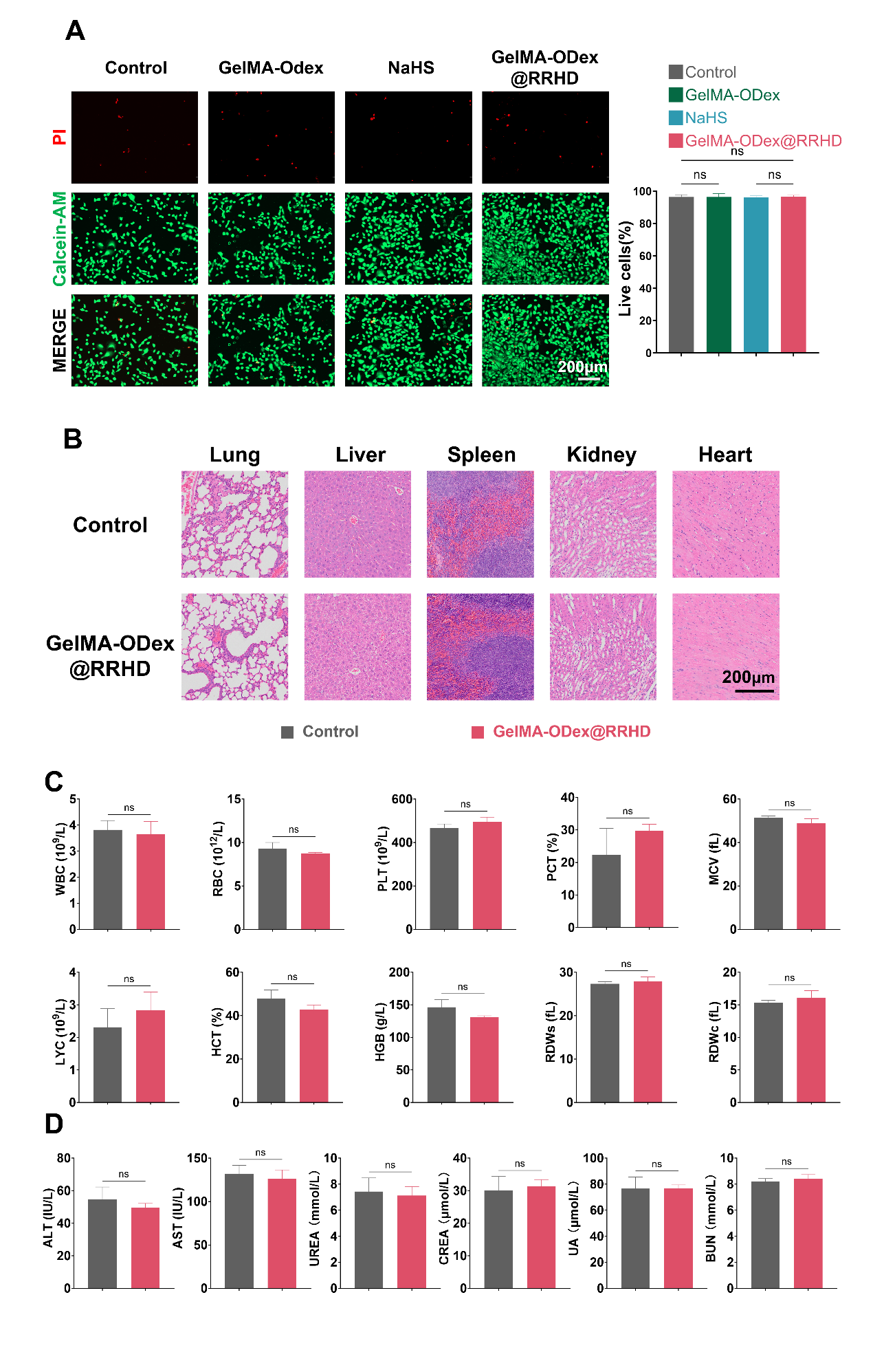
**

Figure S5. Biocompatibility of GelMA-ODex@RRHD.

1. Live/Dead Cell Staining of HUVECs and statistical data of live cells (%). (B) *In vivo* toxicity evaluation of GelMA-ODex@RRHD in major organs. (lung, liver, spleen, kidney, and heart on day 16. (C) Blood analysis on day 16. (D) Serum levels of liver function tests: alanine transaminase (ALT) and aspartate transaminase (AST); serum levels of renal function tests, blood urea nitrogen (BUN), creatinine (CRE) and uric acid (UA) (n=3; mean±s.d.; ns, not statistically significant; *p<0.05, **p<0.01, ***p<0.001, ****p<0.0001)


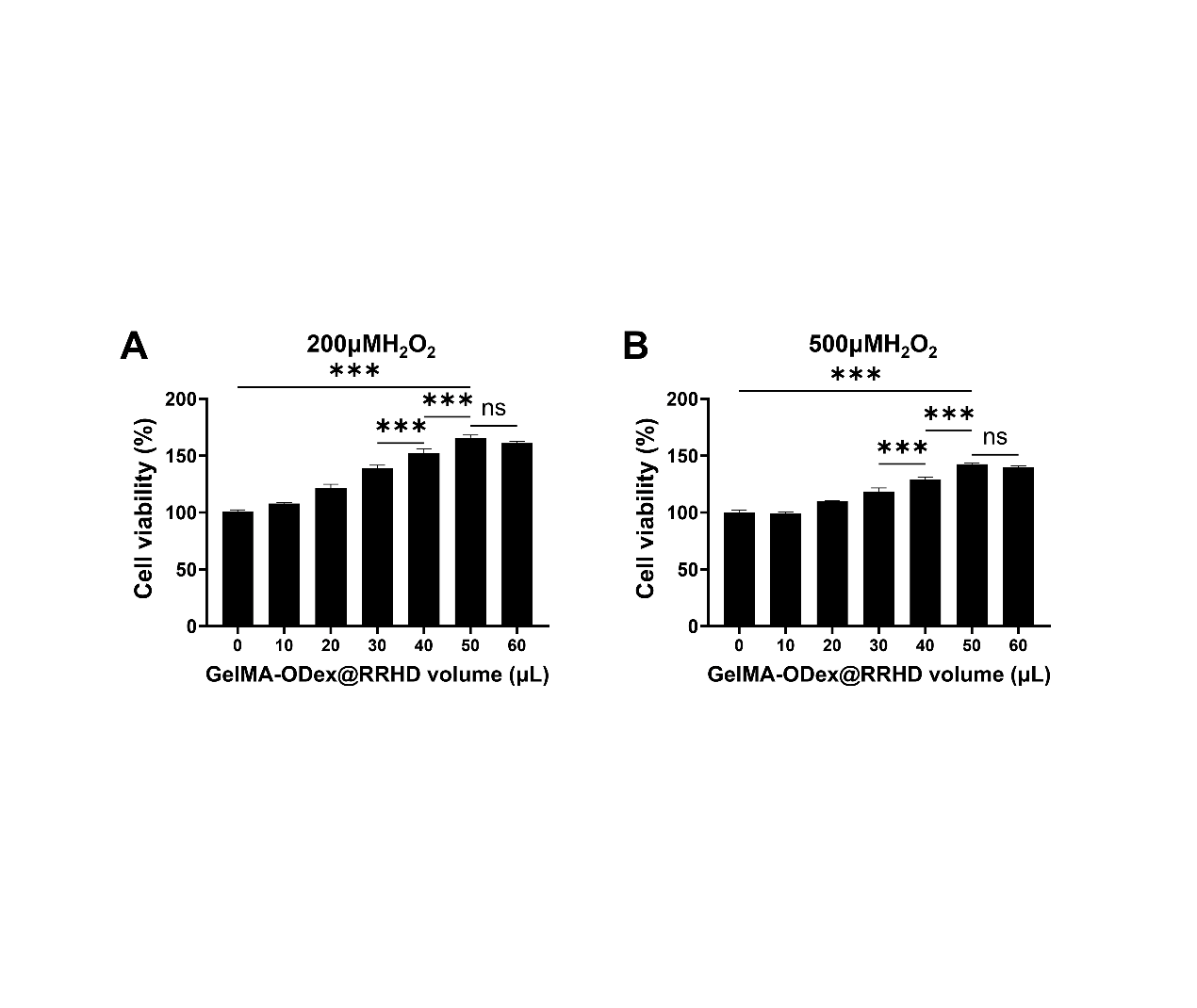
Figure S6. (A, B) Proliferations of HUVECs incubated with GelMA-ODex@RRHD at various volumes with 200 μΜ H_2_O_2_ and 500 μΜ H_2_O_2._ (n=3; mean±s.d.; ns, not statistically significant; *p<0.05, **p<0.01, ***p<0.001, ****p<0.0001)


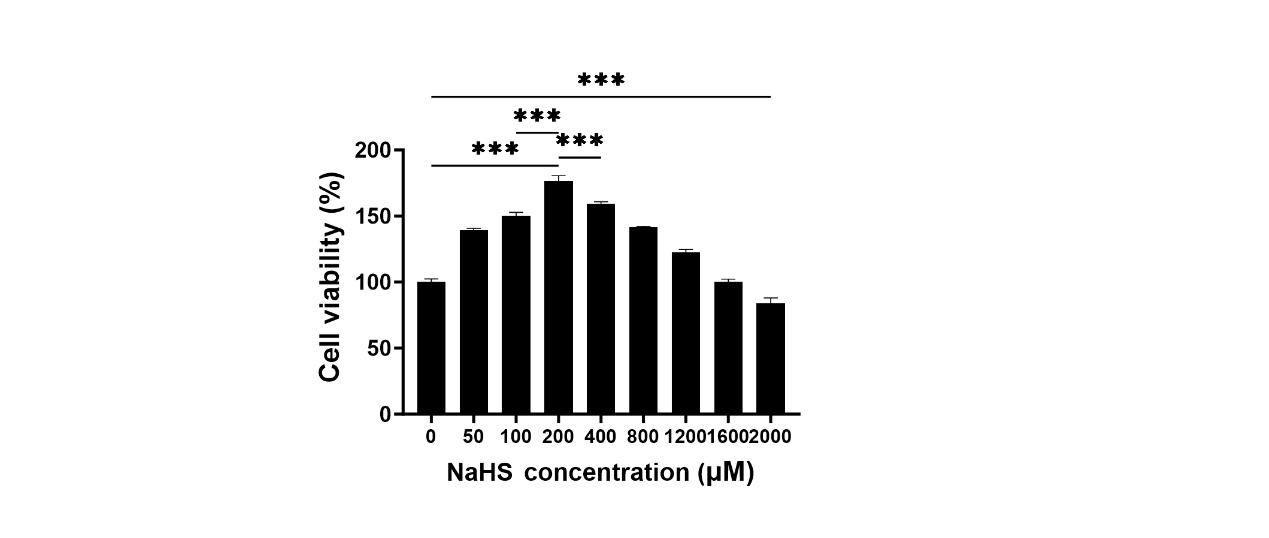


Figure S7. Proliferations of HUVECs incubated with free NaHS at various concentrations. (n=3; mean±s.d.; ns, not statistically significant; *p<0.05, **p<0.01, ***p<0.001, ****p<0.0001)
